# Genetic constraint at single amino acid resolution in protein domains improves missense variant prioritisation and gene discovery

**DOI:** 10.1186/s13073-024-01358-9

**Published:** 2024-07-11

**Authors:** Xiaolei Zhang, Pantazis I. Theotokis, Nicholas Li, the SHaRe Investigators, Euan Ashley, Steven D. Colan, Sharlene M. Day, Adam Helms, Carolyn Y. Ho, Jodie Ingles, Daniel Jacoby, Neal K. Lakdawala, Michelle Michels, Iacopo Olivotto, Anjali Owens, Victoria N. Parikh, Alexandre C. Pereira, Joseph Rossano, Sara Saberi, Chris Semsarian, Samuel Wittekind, Caroline F. Wright, Kaitlin E. Samocha, Nicola Whiffin, James S. Ware

**Affiliations:** 1 National Heart & Lung Institute, Imperial College London, London, UK; 2 MRC Laboratory of Medical Sciences, Imperial College London, London, UK; 3 Royal Brompton & Harefield Hospitals, Guy’s and St. Thomas’ NHS Foundation Trust, London, UK; 4 Department of Clinical and Biomedical Sciences, University of Exeter Medical School, Royal Devon & Exeter Hospital, Exeter, UK; 5 Center for Genomic Medicine, Massachusetts General Hospital, Boston, MA USA; 6 Program in Medical and Population Genetics, Broad Institute of MIT and Harvard, Cambridge, MA USA; 7 Centre for Human Genetics, University of Oxford, Oxford, UK; 8 Big Data Institute, Li Ka Shing Centre for Health Information and Discovery, University of Oxford, Oxford, UK; 9 Present address: European Bioinformatics Institute, Wellcome Genome Campus, Hinxton, UK

**Keywords:** Genetic constraint, Missense variant interpretation, Clinical interpretation, Protein domains, Developmental disorders

## Abstract

**Background:**

One of the major hurdles in clinical genetics is interpreting the clinical consequences associated with germline missense variants in humans. Recent significant advances have leveraged natural variation observed in large-scale human populations to uncover genes or genomic regions that show a depletion of natural variation, indicative of selection pressure. We refer to this as “genetic constraint”. Although existing genetic constraint metrics have been demonstrated to be successful in prioritising genes or genomic regions associated with diseases, their spatial resolution is limited in distinguishing pathogenic variants from benign variants within genes.

**Methods:**

We aim to identify missense variants that are significantly depleted in the general human population. Given the size of currently available human populations with exome or genome sequencing data, it is not possible to directly detect depletion of individual missense variants, since the average expected number of observations of a variant at most positions is less than one. We instead focus on protein domains, grouping homologous variants with similar functional impacts to examine the depletion of natural variations within these comparable sets. To accomplish this, we develop the Homologous Missense Constraint (HMC) score. We utilise the Genome Aggregation Database (gnomAD) 125 K exome sequencing data and evaluate genetic constraint at quasi amino-acid resolution by combining signals across protein homologues.

**Results:**

We identify one million possible missense variants under strong negative selection within protein domains. Though our approach annotates only protein domains, it nonetheless allows us to assess 22% of the exome confidently. It precisely distinguishes pathogenic variants from benign variants for both early-onset and adult-onset disorders. It outperforms existing constraint metrics and pathogenicity meta-predictors in prioritising de novo mutations from probands with developmental disorders (DD). It is also methodologically independent of these, adding power to predict variant pathogenicity when used in combination. We demonstrate utility for gene discovery by identifying seven genes newly significantly associated with DD that could act through an altered-function mechanism.

**Conclusions:**

Grouping variants of comparable functional impacts is effective in evaluating their genetic constraint. HMC is a novel and accurate predictor of missense consequence for improved variant interpretation.

**Supplementary Information:**

The online version contains supplementary material available at 10.1186/s13073-024-01358-9.

## Background

Quantifying the depletion of natural variation in human populations provides a powerful approach to identify variants of large effect [1–8]. Since variants causing severe early-onset disorders are under selective pressure in transmission, they are observed less often than functionally neutral variants. Such depletion of genetic variation (constraint) has been shown to provide strong evidence to prioritise disease-associated genes [1–3], identify critical regions within genes [4, 5], and investigate the effect of non-coding variants [6–8].

However, these existing constraint metrics [1–5] have limited resolution to analyse individual residues, and limited application in genes with sparsely distributed or small percentages of pathogenic missense variants since they explicitly rely on signals clustered linearly within genes (Additional File 1: Fig. S1). To address this issue, we sought to develop an amino-acid level constraint metric. Given that we expect to observe on average one missense variant for every six bases in the exome from the sample size in gnomAD (a total of 5,206,202 missense variants observed out of a 30-Mbp exome size; gnomAD v2.1.1), we are still underpowered to evaluate the depletion of variants at individual residues. Instead, we evaluate homologous residues to aggregate the genetic constraint signal. While previous studies have combined variant information over homologous residues to infer functional effect [9–16], missense variant pathogenicity estimated by genetic constraint within general human populations has not been studied.

## Methods

Here we develop an amino-acid level constraint metric by aggregating the signal over evolutionarily equivalent positions across human protein domains. While there are alternative definitions of homology, we use protein domain families defined by the Pfam database [17], which identifies regions of homology in most genes (see the “ Discussion” section on alternative approaches). Of 70 million possible missense variants (defined by NCBI RefSeq Select transcripts [18]) in the human genome, 28 million are mapped to Pfam protein domain families. After excluding residues with limited statistical power due to a low number of domain copies (see Supplementary Methods), about 16 million missense variants (~ 22% of all possible missense variants) are assessable. In the development and evaluation of HMC scores, we focus on 15,236,101 missense variants that are not common in human populations (minor allele frequency (MAF) < 0.1% or absent from the Genome Aggregation Database (gnomAD) v2.1.1; 125,748 samples) [2].

**Fig. 1:**
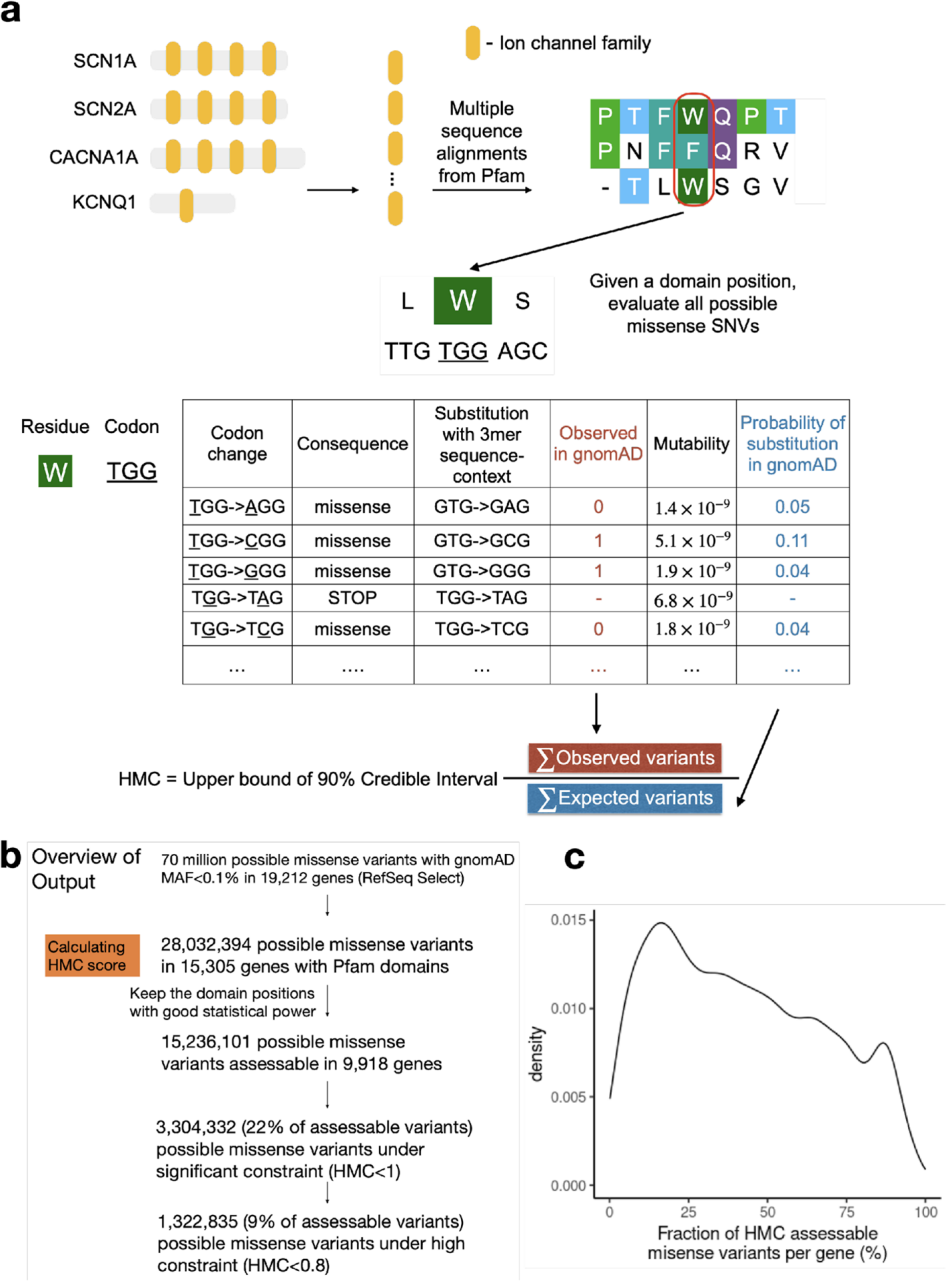
Overview of developing Homologous Missense Constraint. **a** Here, we illustrate how to calculate HMC scores using genes with ion channel domains (Pfam ID: PF00520). Evolutionarily equivalent residues were identified by aligning protein sequences across the protein domain family. Given a domain position with equivalent residues, the observed/expected ratio is calculated to measure the genetic constraint of missense variants at this domain position. HMC score is defined as the upper bound of 90% credible interval of the observed/expected ratio. HMC < 1 indicates significant (*P-*value < 0.05) depletion of missense variation at that residue, and missense variants at these positions are predicted as deleterious. **b** Summary of total number of missense variants evaluated by HMC. **c** Fraction of HMC assessable missense variants across genes (only include 9918 genes with assessable variants). For a few genes, nearly all missense variants are assessable while a few genes have very few assessable variants. For most genes, typically 20–63% variants are assessable (median = 40%)

Given a set of homologous proteins, we calculated the genetic intolerance of missense variants at individual homologous residues. The genetic intolerance score is calculated as the ratio of the number of rare missense variants observed in the 125 K gnomAD population (Observed) to the number of neutral substitutions expected (Expected). The expected number of neutral substitutions is predicted using a mutability model that takes account of tri-nucleotide sequence context, CpG methylation levels, and sequencing coverage, following a null model described previously (Fig. 1; Additional File 1: Fig. S2; Additional File 1: Fig. S3).

Alternative approaches were proposed previously to measure genetic intolerance in sub-genic regions using human population data, including comparing the number of variants from the general population to those observed in patients (e.g. PER [15]), comparing the number of common non-synonymous variants to that of all protein-coding variants (e.g. subRVIS [3, 16]) and calculating the counts of non-synonymous variants over the counts of synonymous variants (e.g. MetaDome [14, 19] and MTR [14, 19]). Our model is used to predict the expectation of neutral variants, which yields improved statistical power compared with alternative genetic intolerance measures that rely on empirical observations [1, 20, 21].

The Homologous Missense Constraint (HMC) score is defined as the upper bound of the 90% credible interval of the Observed/Expected ratio (Fig. 1). A protein residue with the HMC score < 1 indicates that missense variants affecting the homologous residues are significantly depleted compared with expectation, indicating negative selection (*P*-value < 0.05) and that they are likely to be deleterious. 3,304,332 possible missense variants (21.7% of the assessable and rare) impact-constrained residues with HMC < 1. Of these, 1,322,835 (8% of the assessable and rare) occur at highly constrained residues, defined by a more stringent threshold of HMC < 0.8, which we find clinically relevant, as demonstrated in the “ Results” section.

We robustly evaluated the accuracy of HMC scores using a wide range of independent tasks, including (i) assessing the classification performance of HMC using ClinVar [22] variant interpretations as a gold standard; (ii) assessing whether HMC prioritises disease-associated variants using case–control analyses in cohorts of patients with known disease phenotypes, without reliance on a gold-standard variant interpretation as a reference, and (iii) evaluating HMC using variants with in vitro functional altered assays. We further evaluated the ability of HMC to improve variant interpretations by (i) comparing the distribution of HMC-predicted constrained variants with other existing constraint scores, and (ii) testing whether HMC could identify new disease-associated genes.

## Results

### Homologous Missense Constraint precisely identifies pathogenic variants

First, we showed that HMC can distinguish pathogenic variants from benign variants in ClinVar. We note that ClinVar pathogenic missense variants are significantly enriched in Pfam domains (Rate_Pathogenic vs Benign_ = 2.68, 95% CI = 2.63–2.73), and in our defined assessable regions (Pfam domains with multiple copies across the genome) (Rate_Pathogenic vs Benign_ = 2.10, 95% CI = 2.06–2.15) compared with ClinVar benign variants, indicating domains are hotspots for pathogenic missense variants.

**Fig. 2:**
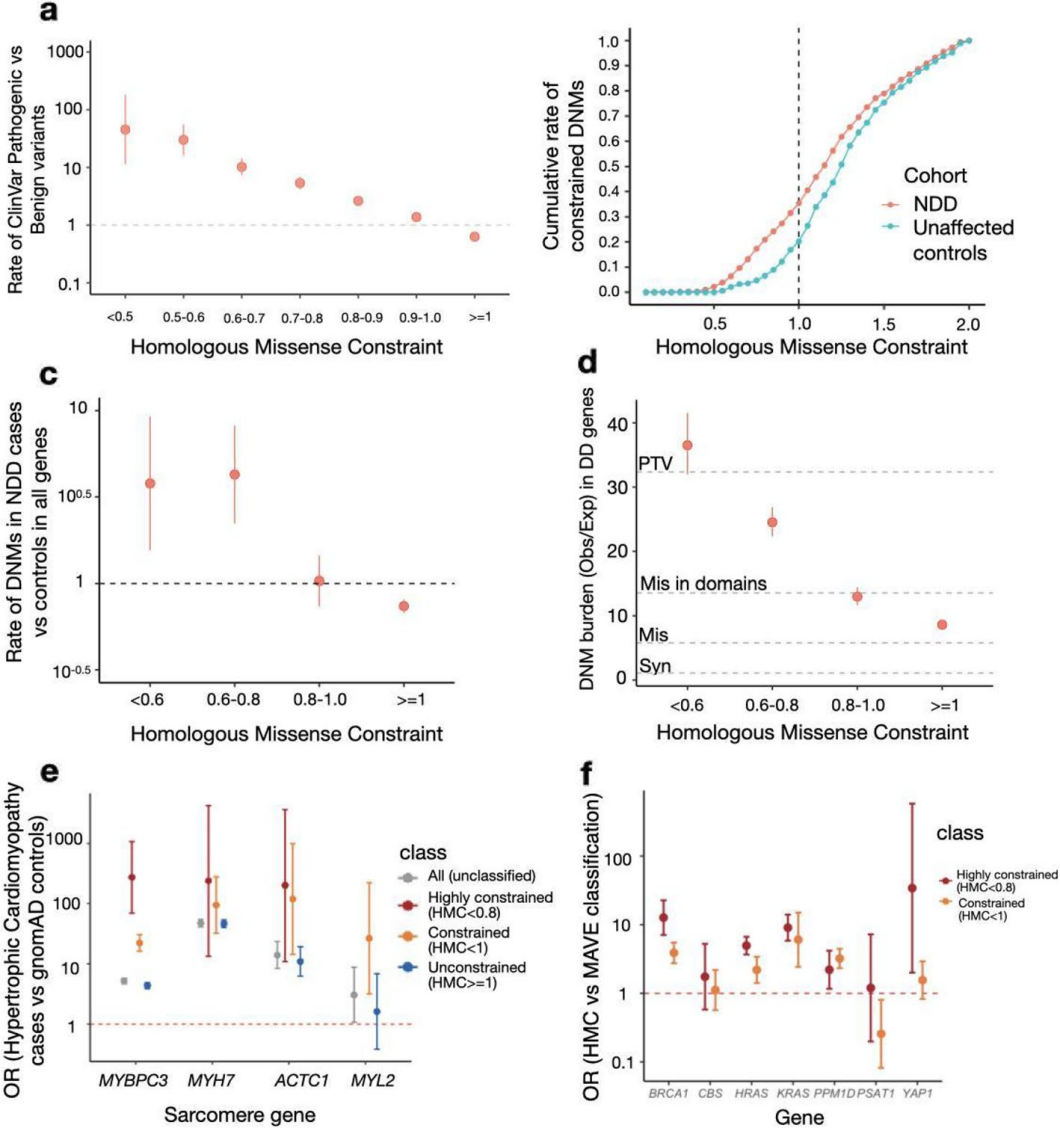
HMC accurately distinguishes pathogenic variants from benign variants. **a** Highly constrained positions within protein domains are enriched for pathogenic variants and unconstrained variants are depleted for pathogenic variants. The rate of ClinVar Pathogenic variants vs Benign variants (risk ratios) within various HMC constrained/unconstrained bins are shown with 95% CI. Rate of ClinVar Pathogenic vs Benign variants (Rate_Pathogenic vs Benign_) was calculated as *N*_in the bin,pathogenic_/*N*_total,pathogenic_ vs *N*_in the bin,benign_/*N*_total,benign_. A total of 13,009 ClinVar Pathogenic and 3914 ClinVar benign variants were assessed. **b** Missense de novo mutations observed in a cohort of individuals with neurodevelopmental disorders (NDD; *n* = 5264) are found at more highly constrained residues than de novo mutations observed in unaffected controls (*n* = 2179). The cumulative rate of constrained de novo mutations in cases (*N*_DNMs with HMC<*X* in cases_/*N*_total DNMs in cases_) was plotted to compare with that in controls (*N*_DNMs with HMC<*X* in controls_/*N*_total DNMs in controls_). In total, 1209 DNMs in cases and 337 in controls are assessed in all genes. **c** Missense de novo mutations affecting highly constrained domain positions are significantly enriched in NDD cases versus unaffected controls. The rate of DNMs in cases was compared with that in controls in HMC-constrained/unconstrained bins. The rate of DNMs in cases vs controls was calculated as *N*_in the bin,cases_/*N*_total,cases_ vs *N*_in the bin,controls_/*N*_total,controls_. **d** In 285 genes associated with developmental disorders, HMC prioritises damaging de novo missense mutations with a comparable effect size as protein-truncating variants (PTV) in 31,058 parent-proband trios of developmental disorders (DD). We compared the prevalence of missense de novo mutations (DNM) in established DD-associated genes in individuals with DD against that of expected de novo mutations predicted by context-based mutability, and plot the ratio (“burden”) for missense DNMs stratified by HMC score. The burden (Obs/Exp) ratio was calculated as N _observed DNMs in the bin, in cases_/N_expected DNMs in the bin, in cases_. As baseline references, the dotted lines show the burden (Obs/Exp) ratio for synonymous DNMs (OR_Syn_ = 1.1, 95% CI = 1.0–1.2), missense DNMs (without HMC stratification; OR_Mis_ = 5.8, 95% CI = 5.6–6.0), missense DNMs within annotated Pfam domains (OR_Mis in domains_ = 13.5, 95% CI = 12.9–14.3), and protein-truncating DNMs (OR_PTV_ = 32.4, 95% CI = 30.8–34.0). Missense DNMs at the most highly constrained residues (HMC < 0.6) show an association signal similar to that of protein-truncating DNMs. **e** Highly constrained (HMC < 0.8) or nominally constrained missense variants (HMC < 1) have increased association with hypertrophic cardiomyopathy compared with controls. We calculated the odds of carrying a rare missense variant for individuals with hypertrophic cardiomyopathy, and for the gnomAD reference population, and show the odds ratio for all rare missense variants, and for rare missense variants stratified by HMC scores. Constrained variants in *MYBPC3* are more strongly disease associated. Data are sparser for the other three genes shown, which are much rarer causes of hypertrophic cardiomyopathy, but the trend is concordant. **f** The association between HMC-constrained missense variants (highly constrained HMC < 0.8 or nominally constrained HMC < 1) and MAVE pathogenic classification in DD-related genes measured by Odds Ratio and its 95% CI. All the seven assessable genes show a positive association with HMC highly constrained variants (HMC < 0.8) and five of them show significant association: *BRCA1*,* CBS*,* HRAS*,* KRAS*,* PPM1D*,* PSAT1* and* YAP1*

Applying HMC within protein domains, we found that ClinVar pathogenic variants are more likely to occur at constrained domain positions (HMC < 1; Rate_Pathogenic vs Benign_ = 3.9, 95% CI = 3.5–4.2, *P*-value = 1 × 10^–304^) compared with unconstrained domain positions (HMC ≥ 1; Rate_Pathogenic vs Benign_ = 0.62, 95% CI = 0.61–0.64, *P*-value = 1 × 10^−311^) (Fig. 2a). The strength of the association increases as domain residues are under stronger genetic constraint (HMC < 0.5; Rate_Pathogenic vs Benign_ = 37.9, 95% CI = 15.7–91.3, *P*-value = 1 × 10^−41^) indicating that variants with lower HMC scores are more likely to be disease-causing.

Next, we asked whether HMC could prioritise deleterious de novo mutations (DNMs). We analysed published DNMs identified in 5264 probands ascertained with severe neurodevelopmental delay (NDD) and 2179 unaffected controls [23]. We found that de novo missense mutations in highly constrained domain positions (HMC < 0.8) are significantly enriched in NDD cases (Rate_NDD cases vs controls_ = 4.1, 95% CI = 2.4–6.9, *P*-value = 3.1 × 10^−10^; Fig. 2b–c). Similarly, highly constrained DNMs are significantly enriched in probands ascertained with autism spectrum disorders (Rate_ASD cases vs controls_ = 2.2, 95% CI = 1.3–3.7, *P-*value = 0.0028; Additional File 1: Fig. S4). In a larger trio cohort with 31,058 probands of developmental disorders (referred hereafter as “the 31 K DD cohort”) [24], we further evaluated the enrichment of constrained DNMs (the ratio of observed to background expectation [20, 25]) in 285 dominant DD-associated genes that showed statistical enrichment of DNMs in that cohort. While missense variants located in annotated domains have a higher burden than those located elsewhere (Obs/Exp = 13.6, 95% CI = 12.9–14.3), HMC can further narrow down to a subset as [20, 25]highly constrained (< 0.8) with an effect size close to that of protein-truncating variants (Obs/Exp = 27.6, 95% CI = 25.5–29.7 vs PTV: Obs/Exp = 32.4, 95% CI = 30.1–34.0; Fig. 2d).

We also tested the ability of HMC to predict deleterious variants causing adult-onset disorders. We performed a case–control gene burden test in 6327 patients with hypertrophic cardiomyopathy from the SHaRe registry [23] using the 125,748 gnomAD v2.1.1 exomes as controls. For four sarcomere genes with HMC assessable variants, cases carry more HMC-constrained variants than controls compared to unconstrained or unclassified variants (Fig. 2e), though this is only individually significant for *MYBPC3* (*P*-value = 1 × 10^−121^), likely due to limited power given a relatively low total number of variants in assessable positions of other genes. We expect HMC to have more power and a narrower confidence interval in genes with domains from a large Pfam family with more assessable positions. As shown by the examples of cardiomyopathy genes, the I-set (Pfam ID: PF07679) and FN3 (Pfam ID: PF00041) domains in *MYBPC3* belong to large domain families with 785 and 597 homologous copies respectively in the exome, while domains from the other three tested genes belong to domain families with fewer than 72 copies in the exome (the largest domain family: EF-hand_1 (Pfam ID: PF00036) in *MYL2*).

As a further independent evaluation, we compared HMC with functional data from multiplex assays of variant effect (MAVEs) obtained from ProteinGym [26]. There are 17 genes that we can both evaluate using MAVE data and HMC scores including 14,813 variants. Across these genes, HMC highly constrained classification (HMC < 0.8) shows a significant association with MAVE functional classification (OR = 1.1, 95% CI = 1.0–1.2) and medium correlation (mean Spearman *r* = 0.19 and mean AUC = 0.59) (Additional File 1: Table S1). For seven genes related to rare monogenic developmental disorders (in the DDG2P panel), HMC has both a stronger association (OR = 1.5, 95% CI = 1.3–1.7) and stronger correlation with MAVE data (mean Spearman *r* = 0.21 and mean AUC = 0.65) on the ranking of variant pathogenicity (Fig. 2f). We infer that the strength of the relationship between the underlying reproductive fitness and the functions measured by MAVE assays influences apparent performance evaluation. Genes under strong negative selection, as exemplified by developmental disorder genes, are expected to have a higher association with HMC (Additional File 1: Fig. S5).

### HMC is highly precise and is complementary to existing metrics to prioritise missense variants

**Fig. 3:**
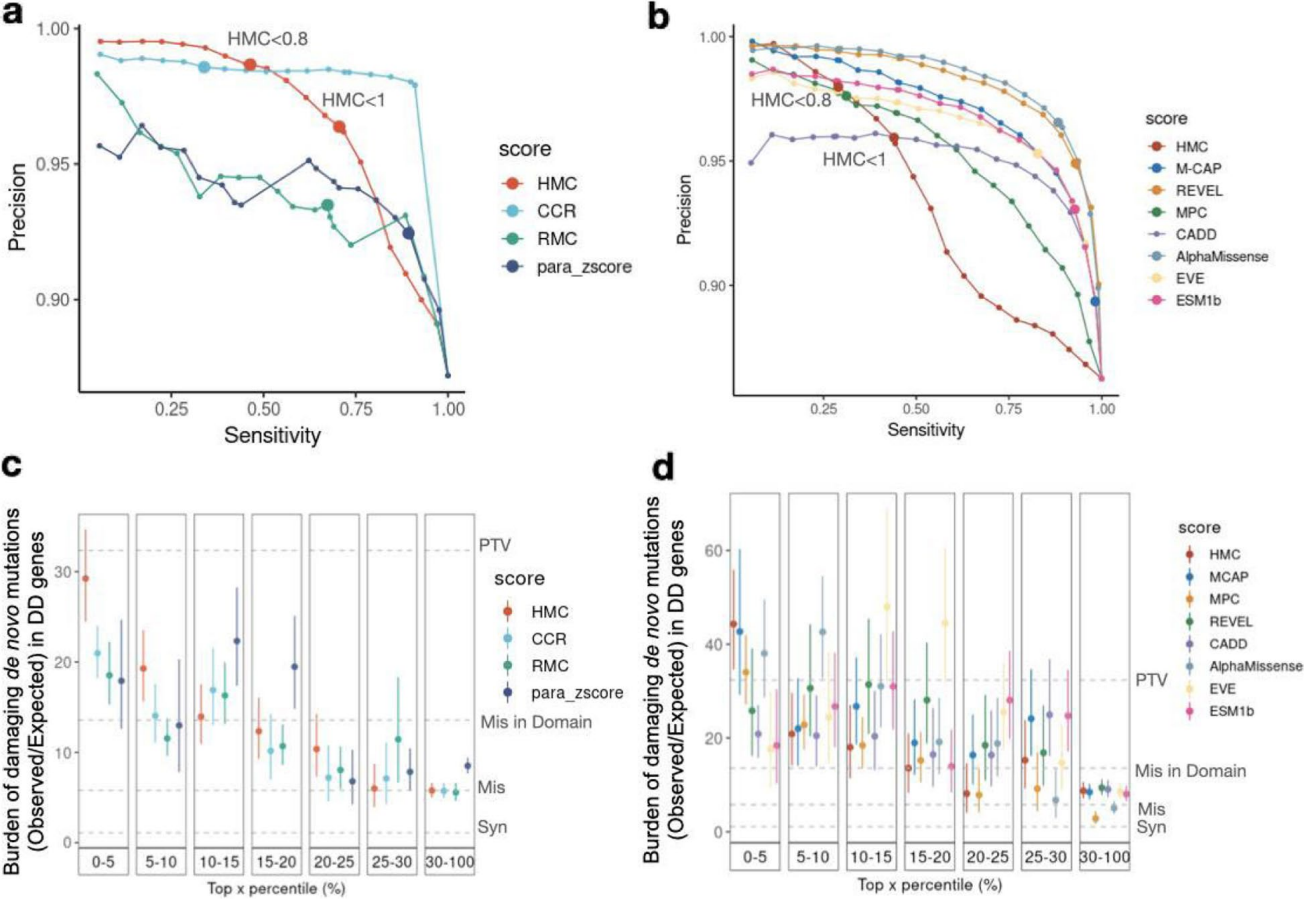
HMC has greater precision than other constraint metrics, and comparable performance to meta-predictor pathogenicity scores. **a** Using ClinVar variants, the precision-sensitivity curve demonstrates that HMC has higher precision over the constraint- and homologous-residue-based methods in top-ranked variants and within authors’ recommended thresholds (dots with larger size). The recommended threshold and the corresponding precision and sensitivity for each tool are HMC < 0.8 (98.6%, 48.8%), CCR > 95 (98.6%, 34%), RMC < 0.6 (93.5%, 67.4%) and para_zscore > 0 (92.4%, 89.3%). **b** HMC has comparable precision as existing state-of-the-art supervised meta-predictors. Dots with larger size indicate the performances (precision, sensitivity) using authors’ recommended thresholds: HMC < 0.8 (98.0%, 29.0%), M-CAP ≥ 0.025 (89.4%, 98.2%), REVEL > 0.5 (94.9%, 92.9%), CADD ≥ 10 (no variants are scored as deleterious), MPC ≥ 2 (97.6%, 31.2%), AlphaMissense ≥ 0.5 (96.5%, 88.3%), EVE ≥ 0.5 (95.3%, 82.8%), ESM1b ≤  − 7.5 (93.1%, 92.8%). **c**–**d** Using the 31 K trio data of DD, the burden of de novo mutations (observed/expected) in DD-associated genes is compared to evaluate the precision of predicting damaging variants (the higher the burden, the more likely the variants are damaging). For a given tool, variants are grouped into bins based on their percentile of predicted pathogenicity among all assessed variants. As baseline references, the dotted lines show the burden ratio for synonymous DNMs, missense DNMs, missense DNMs within annotated Pfam domains, and protein-truncating DNMs as shown in Fig. 2d. **c** Comparison between HMC with existing constraint-based and homologous residue-based scores using DNM burden and its 95% confidence interval; **d** Comparison between HMC with existing state-of-the-art meta-learners using DNM burden and its 95% confidence interval

We next evaluated the performance of HMC against existing pathogenicity scores, using ClinVar variants as a reference set. We first compared HMC to existing sub-genic constraint models that aim to predict deleterious variants without supervised training on known pathogenic variants including Constrained Coding Region [4] (CCR), Regional Missense Constraint [5] (RMC), and a homologous-residue-based conservation metric para_zscore [27]. As each approach generates predictions on different areas of the exome, we analysed the intersection of ClinVar variants that can be scored by all four methods (3661 pathogenic and 537 benign variants). Among HMC, CCR, RMC and para_zscore, CCR has the highest area under the Precision-Sensitivity curve (Fig. 3a). Within the authors’ recommended thresholds of classifying deleterious variants, HMC achieves the highest precision > 98.6% (under the high constraint threshold < 0.8). HMC’s precision is lower for variants with constraint score between 0.8 and 1 as expected, but HMC applied at this more lenient threshold remains the second-best precision among the four scores, with preserved sensitivity. At the threshold for HMC > 1, precision quickly decreases indicating its limited usage in this range.

We also compared HMC to the state-of-the-art machine-learning-based variant pathogenicity predictors: M-CAP [28], MPC [5], REVEL [29], CADD [30], AlphaMissense [31], EVE [31, 32] and ESM1b [33] (Fig. 3b). A set of 9187 ClinVar pathogenic variants and 1465 benign variants that could be assessed by all methods were used to evaluate performance. AlphaMissense performs the best with the highest precision-sensitivity area under the curve among all the tools. Using the high constraint threshold (< 0.8), HMC has the highest precision at the authors’ recommended classification thresholds, with reduced precision (as would be expected) at the nominal constraint threshold (< 1). We note comparable performance, with most tools achieving very high precision (≥ 97%) at the left of the PRC curve. Here we caution that benchmarking against the supervised meta-predictors (including M-CAP, MPC, REVEL and CADD) using ClinVar variants in this context might be biased since they leverage multiple features and previously developed scores, some of which have been trained directly or indirectly on ClinVar, thus potentially leading to an inflated performance of these tools in this evaluation. In contrast, HMC remains independent of the well-established pathogenic variant sets in the score construction and evaluation.

We further compared HMC with the above existing pathogenicity scores for prioritising deleterious DNMs. Using the 31 K DD cohort, HMC outperforms all ten existing tools, being able to prioritise a subset of DNMs with the highest enrichment in the 285 dominant DD genes (top 5%, Fig. 3c and d), with an effect size as strong as protein-truncating variants. This highlights that HMC is highly precise in identifying de novo missense variants causing DD compared with existing approaches.

**Fig. 4:**
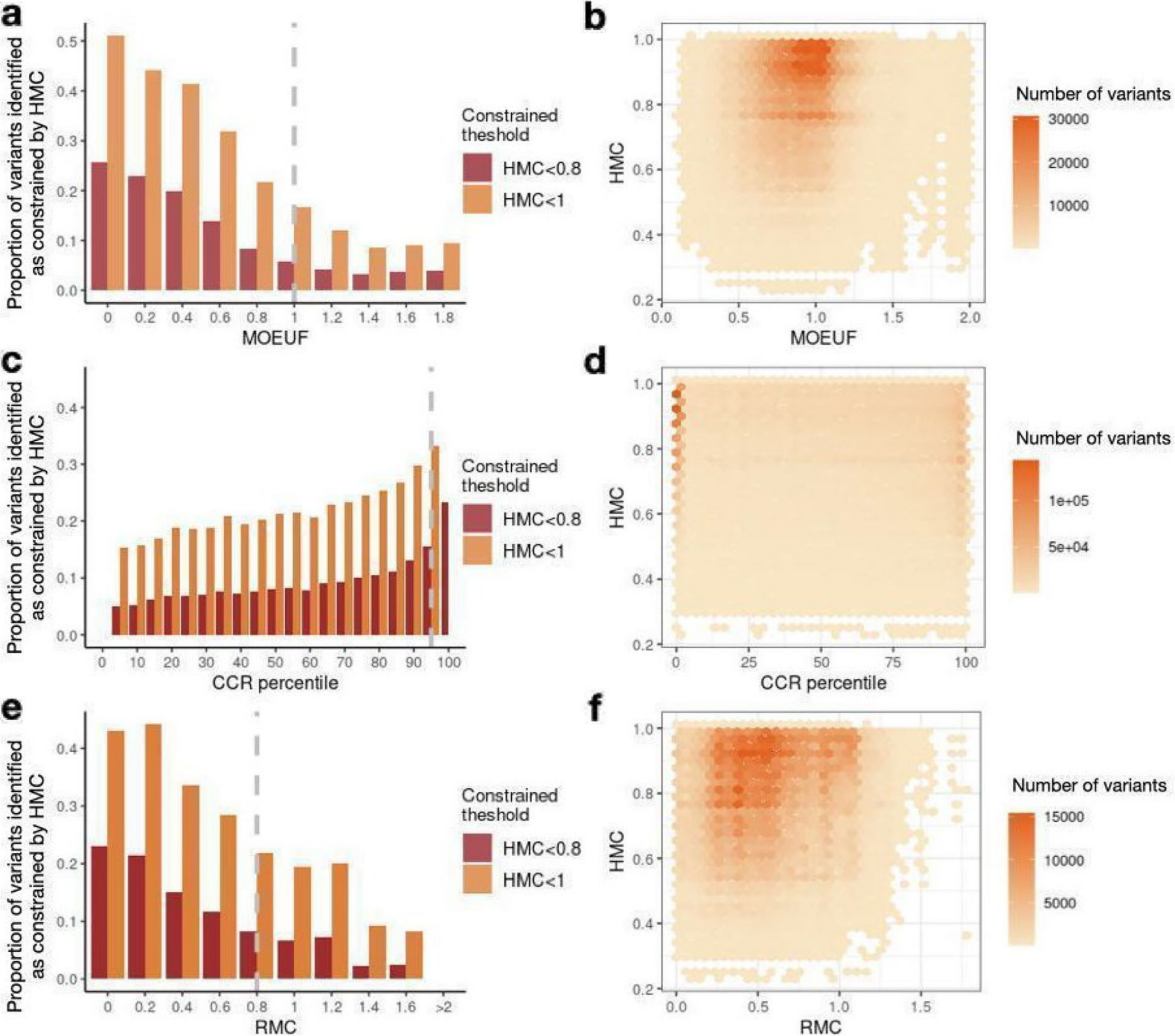
Comparing the distributions of HMC score with existing gene-level and regional-level constraint scores. Here we show that HMC is not co-linear with other metrics, and therefore is likely to provide additional information when used in combination. Bar plots in the first column display the proportion of variants identified as constrained by HMC across genes or sub-genic regions (*N*_HMC-constrained variants in the bin_/*N*_variants in the bin_). Providing a further detailed view of the bar plots, 2D-histogram plots in the second column display the number of HMC-constrained variants within various ranges across gene/region constraint scores. **a**–**b** The relationship between HMC and a gene’s MOEUF score (gene-level constraint of missense variants; a lower value indicates higher constraint). A gene with MOEUF ≥ 1 (grey dashed line) is considered as nominally unconstrained. **c**–**d** The relationship between HMC and CCR (a higher percentile indicates higher constraint). A region with CCR percentile < 95% (grey dashed line) is considered unconstrained recommended by authors [4]. **e**–**f** The relationship between HMC and RMC (a lower value indicates higher constraint). A region with RMC > 0.8 (grey dashed line) is considered unconstrained as recommended by authors

After confirming that HMC has favourable precision compared with existing scores, we ask whether HMC is complementary to them. As a measure of genetic constraint within human populations, HMC is methodologically independent of other lines of computational evidence, such as conservation across species and structural effect predictions. To evaluate the predictive power added by HMC compared with the existing constraint metrics, we assessed the number of HMC-constrained variants that could be missed by existing missense constraint metrics including gene-level constraint (MOEUF) [2], and sub-genic regional constraint scores (CCR and RMC) using the intersection of all possible exome missense variants that could be evaluated by the two scores compared. Constrained homologous residues detected by HMC are distributed across full ranges of these existing metrics in either constrained or unconstrained genes/regions. We find if a gene/region is more constrained as a whole, on average it also has more constrained residues compared to a less constrained gene/region (Fig. 4). However, there are substantial numbers of highly constrained missense variants uniquely classified by HMC (< 0.8): 893,063 not prioritised by either of the sub-genic metrics, of which 351,175 are not prioritised by any of the existing metrics. In summary, HMC provides a novel and independent effect prediction that could be combined with other classes of computational evidence to best interpret variants.

### HMC prioritises newly significant genes associated with developmental disorders

We have analysed distributions of HMC scores across all assessable missense variants in different gene categories, including known autosomal dominant and autosomal recessive disease genes, as well as genes causing developmental disorder (with definite confidence from DDG2P), and genes with high GDI [34] (genes with high missense load in the general population and less likely to be disease-causing). We also included genes with high intolerance of variants measured by gnomAD LOEUF (intolerance of loss-of-function variants; < 0.6) and MOEUF (intolerance of missense variants; < 0.6).

Comparing the median of the HMC distributions, we found genes that are intolerant of missense variants (MOEUF constrained) or loss-of-function variants (LOEUF constrained) and disease-causing dominant genes, which are likely under stronger selective pressure, tend to have variants with lower HMC scores compared to genes with high GDI or autosomal recessive ones (two-sample Brown-Mood median test *P*-value < 2.2 × 10^–16^) (Additional File 1: Fig. S5). This reflects that the distribution of HMC across genes corresponds to the selective pressure acting at the gene level.

We further investigate whether HMC could improve gene discovery in developmental disorders given our above analyses showing that (1) HMC represents an orthogonal measure of variant deleteriousness, (2) is highly accurate in predicting disease-causing DNMs in known DD genes, and (3) genes under strong negative selection have variants with lower HMC scores. HMC prioritises a subset of missense DNMs that show a significant excess burden in the 31 K DD probands, in genes that are not previously known to be associated with DD (HMC < 0.8: Obs/Exp = 1.37 95% CI = 1.25–1.51; Additional File 1: Fig. S6), suggesting its potential to discover unknown DD genes. We updated a gene-specific de novo enrichment test (DeNovoWEST) [18] by incorporating HMC to weight missense variants (see Supplementary Methods) in the 31 K DD cohort. Consequently, we observed an increased DNM burden of up-weighted variants and a decreased DNM burden of down-weighted ones, indicating the improved separation of pathogenic from benign variants after adding HMC to the test (Additional File 1: Table S2). Our upgraded tests identified 286 disease-associated genes in the full cohort, and 97 in those previously undiagnosed (probands who do not carry pathogenic variants in consensus diagnostic genes, as previously defined [24]) at genome-wide significance (Bonferroni adjusted *P*-value < 0.05/(2 × 18,762)).

**Fig. 5:**
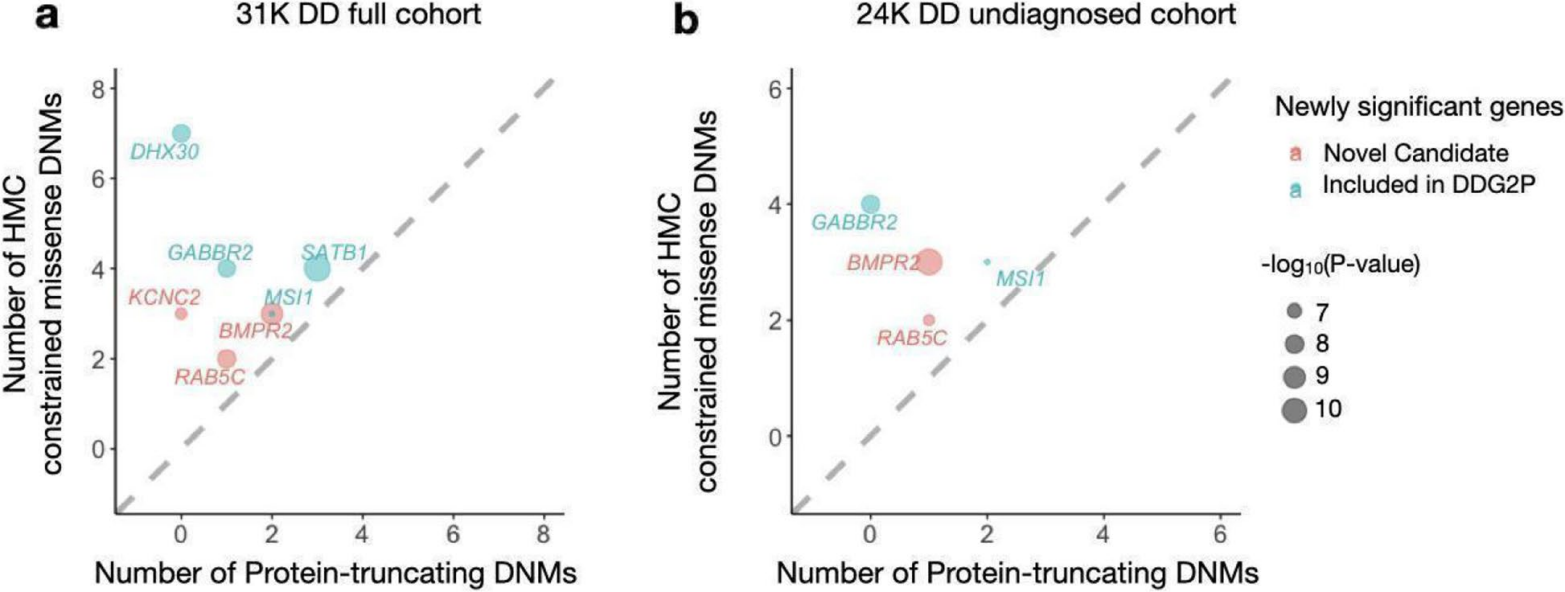
De novo variants identified in 31,058 parent-proband trios reveal seven genes associated with developmental disorders at genome-wide significance for the first time in the full DD cohort (**a**) and the previously undiagnosed subset (**b**). Four of these genes have been previously curated as DD genes on the basis of other lines of evidence, and are already included in the G2P database as established Developmental Disorder genes (blue), while three genes represent new candidate DD genes (red). Numbers of constrained missense DNMs classified by HMC and protein-truncating DNMs were compared. The newly significant associated genes likely act through altered function mechanisms as there are more constrained missense variants than PTVs

Compared with the original study [24], there are seven newly significant genes across the two tests, which carry at least one constrained missense variant, confirming that their elevated significance signal is driven by HMC (Additional File 1: Table S3–S4). Four of these genes have previously been published in association with DD via other lines of evidence and are currently included in the Developmental Disorders Genotype-to-Phenotype Database [35] (DDG2P), indicating that our results provide independent support for their gene-disease association. Three of these genes (*BMPR2*,* KCNC2*, and *RAB5C*) have not yet been included in the DDG2P. *BMPR2* is known to cause pulmonary arterial hypertension [36, 37]. *KCNC2* has been independently suggested to be a new candidate epilepsy gene [38–40]. Importantly, the newly significant genes all have more constrained missense DNMs than protein-truncating DNMs, suggesting the potential involvement of a gene-function-altering mechanism (Fig. 5).

## Discussion

We present HMC, a novel framework to measure missense variant depletion in human populations by grouping variants at equivalent positions of functionally related domains. We have applied our framework to measure the genetic constraint of homologous variants in protein domains. We demonstrate that HMC can precisely identify deleterious missense variants in multiple evaluations. We find that variants at positions scored as highly constrained by HMC (< 0.8) are significantly associated with ClinVar pathogenic classification and in vitro functional classification in the case of *BRCA1* variants. They are also enriched in cases of early-onset disorders and adult-onset disorders exemplified by hypertrophic cardiomyopathy.

Compared with existing established approaches to predict variant effects, HMC directly models the degree of variant depletion in human populations. This eliminates the need for training on labelled datasets to classify variants and prevents circularity that could result in overestimated prediction performance. Compared with existing constraint metrics that aggregate variants over “horizontal” regions of the genome, HMC considers “vertical” space across homologous regions, enabling us to assess genetic constraint with single amino-acid resolution. In the benchmarking using ClinVar variants, HMC outperforms the other constraint and homologous-residue-based methods using the authors’ recommended classification thresholds, indicating the effectiveness of our novel framework to evaluate genetic constraints. Despite that the meta-predictor pathogenicity scores could have inflated performances in classifying ClinVar variants, we still find the precision of HMC remains comparable to them and even outperforms MPC and CADD within the authors’ recommended thresholds. Using de novo variants that are associated with DD, HMC clearly outperforms all the benchmarked existing scores to identify a subset of predicted deleterious missense variants most strongly associated with diseases. Furthermore, we demonstrate that HMC is complementary to the existing methods and enhances our ability to interpret missense variants when used in combination with them. We provide empirical validation, where the integration of HMC into DeNovoWEST identifies seven newly significant genes associated with developmental disorders.

Given its high positive predictive value, we propose that HMC could be used as a constraint metric applied through PP2 following ACMG guidelines in clinical variant interpretation (PP2: “Missense variant in a gene that has a low rate of benign missense variation and where missense variants are a common mechanism of disease” [41]). Within the existing mutational constraint scores, missense constraint at the gene or regional level (gnomAD MOEUF and CCR) has been shown to provide supporting evidence of pathogenicity through the PP2 criterion within the ACMG clinical interpretation framework [42]. Since HMC has higher precision over these gene-level or regional-level constraints, we recommend evaluating PP2 by using HMC first where possible (activating PP2 if HMC < 0.8) before applying gene/region-level constraint as illustrated in Additional File 1: Fig. S7. To combine HMC with machine learning pathogenicity predictors or other lines of evidence, one can follow the rules for combining criteria to classify variants in the ACMG guidelines [41, 43]. Since unconstraint indicates a lack of evidence of pathogenicity, we do not recommend applying HMC unconstrained prediction as evidence of benign impact.

We also demonstrated the structural impact of highly constrained variants using an example of I-set domain (PF07679). The most constrained position by HMC in I-set domain is located at a tryptophan residue (meta position 239 in I-set domain family, HMC = 0.608). This position is also highly conserved across species. For proteins with I-set domains, we illustrated its structural effect using a known pathogenic variant in this position, specifically the TTN missense variant Trp976Arg (NP_001254479.2:p.Trp976Arg) causing dilated cardiomyopathy. Since no experimental structure is available for the corresponding I-set domain in TTN, we used AlphaFold [44] to predict the domain structure. With the predicted structure, we employed missense3D [45] to estimate the structural impact of the amino acid change on the constrained position (Additional File 1: Fig. S8). This amino acid change is predicted to be structurally damaging because a hydrophobic, uncharged tryptophan buried in the core is replaced with a hydrophilic and charged residue, likely disrupting the domain stability. For highly constrained variants, we recommend combining structural analysis to interpret the impact on protein structure and identify potential disease-causing mechanisms.

We have also explored alternative approaches to develop HMC. In this manuscript, we have applied our framework in protein domains. Since there is no gold-standard definition for homologous amino acids, our choice is largely limited by the availability of data. We used protein domain alignment to define homologous residues because there are more genes annotated with protein domains compared with paralogous alignment and structural alignment. Of 19,212 genes included in RefSeq Select, 15,305 have Pfam domains while 14,772 genes have paralogs. For structurally-aligned residues, to the best of our knowledge, there are not yet standard resources publicly available. Given that only ~ 58% of residues in human proteins have confident structural models including experimentally determined structures and homology-based predicted structures [44], the number of residues that could be reliably structurally aligned with others would be even less. The recent radical development of protein structure prediction might also help us to define structurally aligned variants and provide an alternative definition of homologous variants thus increasing the precision of HMC. During the preparation of this manuscript, the coverage of Pfam to annotate protein families has also been significantly expanded by deep learning models, adding functional predictions for 360 human reference proteome proteins without previous Pfam annotations (including the Pfam version we used) [38]. These recent developments would help to increase the statistical power of HMC thus allowing us to evaluate more missense variants. Hence one could revisit this comparison when the size of the datasets goes up in the future.

As the performance of HMC could be affected by the multiple sequence alignment, we also explored whether taking account of the genetic constraint of surrounding amino acids could improve the performance, since the true homologous residues might be expected to lie in neighbouring columns if not directly aligned with each other. Our sensitivity analysis shows that adding more surrounding amino acids could improve sensitivity but compromise precision since there could be more non-relevant residues simultaneously added to dilute the signal (Additional File 1: Fig. S9). To favour precision over sensitivity, we have not included surrounding amino acids in the metric reported here.

Additional features of residues could be added to improve the precision of HMC, such as interspecies conservation and biochemical properties of aligned amino acids. As homologous residues based on sequence might not be always functionally homologous to each other, the performance of HMC could be also affected by exceptions when certain individual residues might have different functional consequences/specifications than homologous residues in their family. Though we chose to keep HMC orthogonal here without adding existing molecular evidence, there is potential for development and improvement by combining HMC with additional features of residues.

We have noticed that HMC has a relatively lower sensitivity compared with the existing methods. There could be several reasons: (a) as we aggregate the genetic intolerance signals across genes, the signals from early-onset disease genes might be diluted if most of the variants in a domain family play roles in late-onset phenotypes. Thus as a group, the residues are not constrained on average, limiting the sensitivity of HMC; (b) as we use the upper limit of the 90% credible interval of constraint measure (95% quantile), an insignificant constraint sore (> 1) could also be caused by limited statistical power because of the low number of homologous variants we can assess as a group. To potentially differentiate the two scenarios for a given domain position, we could use the maximum likelihood estimate (MLE) of constraint value, which is calculated as Obs/Exp. If a domain position with an HMC score (95% quantile) > 1 but MLE < 1, it indicates that the position could be constrained but we do not have enough sample size to evaluate it confidently. For positions with both 95% quantile and MLE > 1, it indicates that there is no evidence that this position is constrained. Our data release has also provided the MLE to help users to interpret the classification.

Looking forward, HMC is a promising framework to evaluate missense variant effect and its statistical power will be increased with the ongoing growth of large-scale population genomics data alongside the developments of annotating the structural effect of variants using deep learning models.

## Conclusions

Here we have described a novel framework to measure genetic intolerance, HMC, to predict deleterious missense variants by aggregating variation at homologous residues. HMC provides a powerful new tool for the interpretation of genetic variation in protein domains.

### Supplementary Information

Additional file 1. Supplementary material including supplementary methods, supplementary figures and supplementary tables.

## Data Availability

The essential scripts used to generate HMC scores and recreate the figures in the main text are available at https://github.com/ImperialCardioGenetics/homologous-missense-constraint. HMC scores for all assessable variants in genome build GRCh37 and GRCh38 are both available via the following portals: (a) www.cardiodb.org/hmc; (b) in UCSC Genome Brower “Constraint scores” track, https://genome.ucsc.edu/cgi-bin/hgTrackUi?hgsid=1670457104_ELF336Rha15ZXZa4xS7BhOCX84dR&c=chr12&g=hmc. External data used in the study were listed in the “Resource Availability” section of the Supplementary Materials.

## Abbreviations

CCR: Constrained Coding Region
DNM: De novo Mutations
DD: Developmental disorders
DDG2P: Developmental Disorders Genotype-to-Phenotype Database
DeNovoWEST: De novo Weighted enrichment simulation test
gnomAD: Genome Aggregation Database
HMC: Homologous Missense Constraint
LOF: Loss-of-function
MAF: Minor allele frequency
MAVE: Multiplexed assay of variant effects
MOEUF: Missense observed/expected upper bound fraction
NDD: Neurodevelopmental delay
Obs/Exp: Number of observed variants/number of expected variants
OR: Odds ratio
PPV: Positive predictive value
PTV: Protein-truncating variants
RMC: Regional missense constraint
