# Supplementary material for: Genetic constraint at single amino acid resolution in protein domains improves missense variant prioritisation and gene discovery
Source: Genome Med. 2024 Jul 11;16:88. doi: 10.1186/s13073-024-01358-9 (PMC11238507; doi:10.1186/s13073-024-01358-9)
Supplement: Supplementary file 1 — Additional file 1. Supplementary material including supplementary methods, supplementary figures and supplementary tables. [file 13073_2024_1358_MOESM1_ESM.docx]

**Supplemental Contents**

[**Supplementary Methods 2**](#_17dp8vu)

[Identification of homologous residues from domain family alignments 2](#_3rdcrjn)

[Annotation of molecular consequences of variants 2](#_26in1rg)

[Developing a selection-neutral, sequence-context mutational model 2](#_lnxbz9)

[Estimating Homologous Missense Constraint 4](#_oy5tro7opn87)

[Evaluating the pathogenicity of ClinVar variants 6](#_1ksv4uv)

[Evaluating the pathogenicity of de novo variants 7](#_44sinio)

[Testing improving power of gene discovery 9](#_3j2qqm3)

[**Supplementary Figures 12**](#_izresmf09zh7)

[**Supplementary Tables 22**](#_35nkun2)

##

##

## **Supplementary Methods**

### **Identification of homologous residues from domain family alignments**

The family alignments of all 6,196 human protein domains generated using the NCBI RefSeq sequence database were downloaded from Pfam[^1^](https://paperpile.com/c/ujbZ45/acs6Q) (Pfam-A.full.ncbi.gz version 32.0). Given a multiple sequence alignment of a domain family, amino acids in the same column of the alignment were considered homologous.

### **Annotation of molecular consequences of variants**

RefSeq Select transcripts were used throughout the whole analysis such that each protein-coding gene has a single high-quality representative transcript. The consequences of variants were annotated by VEP (release 101)[^2^](https://paperpile.com/c/ujbZ45/dsXyo). Only single-nucleotide variants with VEP annotated as “missense_variant” were included in the analysis.

### **Developing a selection-neutral, sequence-context mutational model**

To estimate the number of substitutions expected on a single nucleotide, we constructed a neutral mutational model using the gnomAD reference population. Previous studies have shown that the mutation rate of single nucleotide substitution under neutral selection could be predicted based on sequence context and methylation level[^3^](https://paperpile.com/c/ujbZ45/j9ZTu). Given the baseline substitution rate using a tri-nucleotide sequence text model estimated from variants in intergenic or intronic regions by gnomAD[^4^](https://paperpile.com/c/ujbZ45/m5j7I), we calibrated the baseline mutation rate to probabilities of synonymous variants (presumed to be neutral substitutions) within the 125,478 exomes in gnomAD following the procedures described in the gnomAD flagship paper[^4^](https://paperpile.com/c/ujbZ45/m5j7I).

We firstly used linear regression to predict the proportions of neutral substitutions given the baseline mutation rates. For each possible tri-nucleotide sequence context, the proportion of neutral substitutions is calculated as the ratio of observed synonymous substitutions over all possible synonymous substitutions. For example, to calculate the proportion of neutral substitutions from AAT to AGT, we firstly find the number of all possible synonymous variants introduced by mutating AAT to AGT along the exome and then count those observed in gnomAD v2 exome data. This ratio of “observed” to “all possible” is used as the dependent variable in linear regression. Since the observation of substitutions would be biased by sequencing coverage, at this step only sites with high coverage (median depth 40) are included in the regression. Two linear regression models were fitted, one for substitutions at CpG sites and the other for non-CpG sites (**Fig. S2**). The methylation data for CpG sites was downloaded from gnomAD public datasets and was categorised into three bins: low, medium, and high methylation levels as previously described[^4^](https://paperpile.com/c/ujbZ45/m5j7I). With these predicted probabilities of substitutions, we can estimate the expected number of single-nucleotide variants under neutral selection (Expected) in the 125,478 exomes in gnomAD.

Secondly, we adjusted the probabilities of neutral substitutions for low-coverage sites (median depth<40). To this end, the Observed/Expected ratios for synonymous variants were aggregated for each sequencing coverage (measured by median coverage among gnomAD samples). Given a sequencing coverage, it was calculated as: the expected number of variants is the sum of predicted proportions of neutral substitutions for each site derived from the first step, indicating the number we expect with high-coverage sequencing; the observed number of variants is the sum of observed synonymous variants for each site. A linear model was fitted to predict the Observed/Expected ratios given a sequencing coverage on a log_10_ scale (*R^2^*=0.96, *P-value*<2.2×10^-16^; **Fig. S3)**. The predicted Observed/Expected ratios by the model were used as correction factors to adjust the expected number of variants at low-coverage sites.

### **Estimating Homologous Missense Constraint**

An overview of measuring Homologous Missense Constraint is illustrated in **Figure 1a**. For an aligned position in a Pfam domain family, we assessed all possible missense substitutions. Among all the possible missense substitutions, the number of substitutions directly observed in gnomAD was counted (Observed). The expected number of missense substitutions was calculated as the sum of predicted probabilities of substitutions given by the neutral mutational model (Expected). The genetic intolerance of this aligned position was calculated as the ratio of Observed/Expected.

Given the variability in the number of aligned domains, to control the quality of assessing genetic constraint in homologous residues, we excluded any domain position with less than three expected variants as the number of possible missense variants that occurred at this residue position is too small for us to evaluate genetic constraint robustly. If the number of observed substitutions follows a Poisson distribution under the null hypothesis (no selection), even with zero observed substitutions, the expected number needs to be at least three to reach the significance threshold (the probability of observing zero occurrences with mean occurrence as three is 0.049. In R, it is calculated as “ppois(0,3)=0.049”). It might also indicate the corresponding column is constructed with low confidence filled with a large proportion of gaps (>95% in our observation). Filtering these columns might also limit the effect of alignment bias on defining homologous residues.

Homologous Missense Constraint is defined as the upper limit of 95% confidence interval for the Observed/Expected ratio. The confidence interval for the Observed/Expected ratio was estimated using a Bayesian approach as previously described[^4^](https://paperpile.com/c/ujbZ45/m5j7I). The unknown true Observed/Expected ratio (constraint) was considered as a random variable with a uniform prior between 0 and 2 (in computing, discretized into a sequence of 2000 values from 0 to 2, incremented by 0.001). The likelihood function for a given constraint value is given as the Poisson density:

$Pr \left( X=Observed|constraint=\lambda\right) =\frac{({\lambda*Expected)}^{Observed}e^{-\lambda*Expected}}{Observed!}$.

Thus, the posterior probability of a given constraint value could be derived by:

$Pr\left( constraint=\lambda| Observed, Expected \right)=\frac{Pr \left( constraint=\lambda\right) *Pr(X=Observed|constraint=\lambda)}{\sum\lambda*Pr \left( constraint=\lambda\right) *Pr(X=Observed|constraint=\lambda)}$.

We could further obtain the 90% credible interval of constraint by taking the 5% and 95% quantile from its posterior probability distribution. Therefore, the upper bound of 90% credible interval is taken as the constraint score of homologous residues (HMC). If a residue is scored as HMC <1, it indicates that missense variants disrupting the given domain position are significantly (*P*-value < 0.1) depleted of variants thus under selection pressure.

There are 28,032,394 (~40% of 70 million possible missense variants) missense variants in 15,305 genes (out of 19,212 genes) overlapping 5,807 Pfam domains. After excluding domain positions with limited statistical power, there are 15,236,101 possible missense variants with MAF<0.1% from 699 Pfam families with 78,070 domain positions assessable in 9,918 genes. We identified 3,304,332 possible missense variants (21.7% of assessable) at constrained (HMC<1) positions in 596 Pfam domains. 1,322,835 possible missense variants (8% of assessable) were identified at highly constrained residues (HMC<0.8) of 458 domains (also see summary in **Figure 1b**).

### **Evaluating the pathogenicity of ClinVar variants**

The association of HMC with known disease-causing variants was tested using ClinVar[^5^](https://paperpile.com/c/ujbZ45/1Ne5a). The VCF file was downloaded from the ClinVar public FTP site (version 20201114, https://ftp.ncbi.nlm.nih.gov/pub/clinvar/vcf_GRCh37/archive_2.0/2020/clinvar_20201114.vcf.gz). There are 35,672 unique missense variants classified as Pathogenic in ClinVar, whose clinical significance was recorded as “Pathogenic”, “Likely_pathogenic” or “Pathogenic/Likely_pathogenic”. Variants with recorded conflicting pathogenicity evaluation were excluded. We extracted 22,886 Pathogenic/Likely pathogenic missense variants in Pfam domains. 7,137 Benign/Likely benign variants in Pfam domains were extracted with clinical significance recorded as “Benign”, “Likely_benign” or “Benign/Likely_benign”. After keeping the HMC assessable domain positions, 13,009 Pathogenic/Likely pathogenic and 3,914 Benign/Likely benign variants were used as test data. Only variants with no conflicting interpretation were included in the test set.

The enrichment tests of ClinVar pathogenic missense variants in the assessable region and Pfam domains are conducted in R using: “riskratio(13009,35672-13009,15000000,70000000-15000000)” and “riskratio(22886,35672-22886,28000000,70000000-28000000)”.

### **Evaluating the pathogenicity of *de novo* variants**

To test the enrichment of *de novo* variants (DNMs) prioritised by HMC in affected individuals versus unaffected individuals, we analysed the published DNMs in 5,264 patients ascertained with neurodevelopmental disorders, 6,430 patients ascertained with autism spectrum disorder, and 2,179 unaffected controls curated by Satterstrom *et.al*[*^6^*](https://paperpile.com/c/ujbZ45/hrQrc).

We applied an independent approach to measure the accuracy of predicting damaging missense *de novo* mutations by testing the enrichment of DNMs prioritised by HMC in affected individuals versus neutral variants estimated by a null sequence-context based *de novo* mutational model[^7^](https://paperpile.com/c/ujbZ45/Myl9r). This measurement can also be used to assess whether HMC could distinguish pathogenic and benign variants within disease genes as the enrichment of DNMs in cases vs control individuals can be driven by gene-level disease association. We analysed the published DNMs in 31,058 patients with developmental disorders. The burden of DNMs was calculated as the ratio of the number of observed DNMs to the number of expected DNMs. The number of observed DNMs was directly counted from the variants seen in the cohort. The number of expected DNMs under neutral selection for the cohort is calculated by summing the product of the trinucleotide *de novo* mutation rate and the number of exome samples (2×31,058) for each nucleotide. The *de novo* mutation rate was downloaded from the GitHub repository of denovolyzeR[^8^](https://paperpile.com/c/ujbZ45/7CTXj) (https://github.com/jamesware/denovolyzeR-ProbabilityTables/blob/master/data-raw/fordist_1KG_mutation_rate_table.txt). The effective sample size for X-chromosome is adjusted considering sex-chromosome transmission as previously described[^9^](https://paperpile.com/c/ujbZ45/5RX20). We calculated the 95% confidence interval of the DNM burden, which only depends on the 95% confidence interval for the mean number of observed DNMs. We could estimate it by using an exact method. In R, it is calculated as “poisson.test(n_obs, conf.level=0.95)”.

To be noticed, the set of DNMs published in Satterstrom *et.al*[*^6^*](https://paperpile.com/c/ujbZ45/hrQrc) was a compilation of DNMs from previous publications. Since Satterstrom *et.al*[*^6^*](https://paperpile.com/c/ujbZ45/hrQrc) have harmonised the quality control for 5.2K cases and 2.2K unaffected controls thus we used this dataset for DNM case-control analysis. We used the larger set of 31K cases in the DNM enrichment analysis. Of note, the 5.2K cases are a subset of 31K cases[^6,9,10^](https://paperpile.com/c/ujbZ45/5RX20+hrQrc+FX0wS) but we think this won’t affect the validity of our results: the controls are different in the two analyses. 5.2K is compared with unaffected individuals while 31K is compared with DNM null model. Therefore, even if we fully reuse the same cases, these two analyses shall be considered as independent validations.

### **Testing improving power of gene discovery**

To demonstrate the utility of applying HMC to discover more disease genes reliably, we upgraded the gene-specific *de novo* weighted enrichment simulation test (DeNovoWEST)[^9^](https://paperpile.com/c/ujbZ45/5RX20) by adding HMC to score missense variants. In the original framework of DeNovoWEST, the weight of a missense variant used in the simulation test depends on the regional missense constraint (RMC). Here we incorporated HMC into this framework through the following procedures: (1) combining HMC with regional missense constraint to label constrained missense variants, thus a missense variant is considered as constrained if either RMC or HMC (HMC<0.8) score it as constrained. Compared with the original version, there are 732,404 more missense variants classified as constrained; (2) updating the weights of missense variants used in DeNovoWEST: we calculated the burden of *de novo* missense variants against a null *de novo* mutational model[^7^](https://paperpile.com/c/ujbZ45/Myl9r) and inferred the corresponding positive predictive values (PPV) for all possible categories using constraint (based on step 1) and CADD scores. The newly derived PPV is used as weights in the downstream gene-specific test. The upgraded test was applied separately in the full (n=31,058) and undiagnosed (n=24,288) cohort of parent-proband trios of developmental disorders[^9^](https://paperpile.com/c/ujbZ45/5RX20). Compared with the original version, the DNM burden of constrained missense variants have increased in the 31K DD cohort while the one of unconstrained missense variants have decreased (**Table S2**). This suggests that HMC has improved the classification of pathogenic and benign missense DNMs in the cohort.

We define newly-significant associated genes driven by HMC scoring that meet all the following criteria: (1) only reached genome-wide significance threshold (Bonferroni adjusted *P*-value<0.05/ (2⨉18,762)) in our upgraded test; (2) carries at least one highly constrained missense variant prioritized by HMC (HMC<0.8). In our upgraded tests, there are seven genes in total that reached genome-wide significance level but did not in the original DeNovoWEST tests: *BMPR2, DHX30, GABBR2, KCNC2, MSI1, RAB5C, SATB1* (**Table S3-S4**). All of them have at least one HMC constrained missense variant thus are defined as newly-significant associated genes in our analysis. There are three genes (*ARHGEF9, SETD1B* and *CNKSR2)* that were significant in the original tests but did not in the upgraded tests. Since the significance levels of these three genes before and after the upgrade are in the same scale, we think it’s likely due to random variations in different runs of simulations. For those currently not included in DDG2P, we define them as novel candidate genes associated with DD including *BMPR2, KCNC2* and *RAB5C.*

**Evaluating HMC with MAVE assays**

We compared HMC scores with functional effects measured in multiplex assays of variant effect (MAVEs) curated by the ProteinGym[^11^](https://paperpile.com/c/ujbZ45/UGpd0). There are 29 genes for which we had both MAVE data and HMC scores. To evaluate the accuracy of HMC constrained prediction, we focused on the 17 genes (14,813 variants in total) with HMC positive prediction, acknowledging the limited sensitivity of HMC. There is one gene (*KRAS*) with two available assays measuring different phenotypes, we opted for the one showing the highest correlation with HMC assuming the use of assays would most reflect reproductive fitness effects.

##

## Supplementary Figures


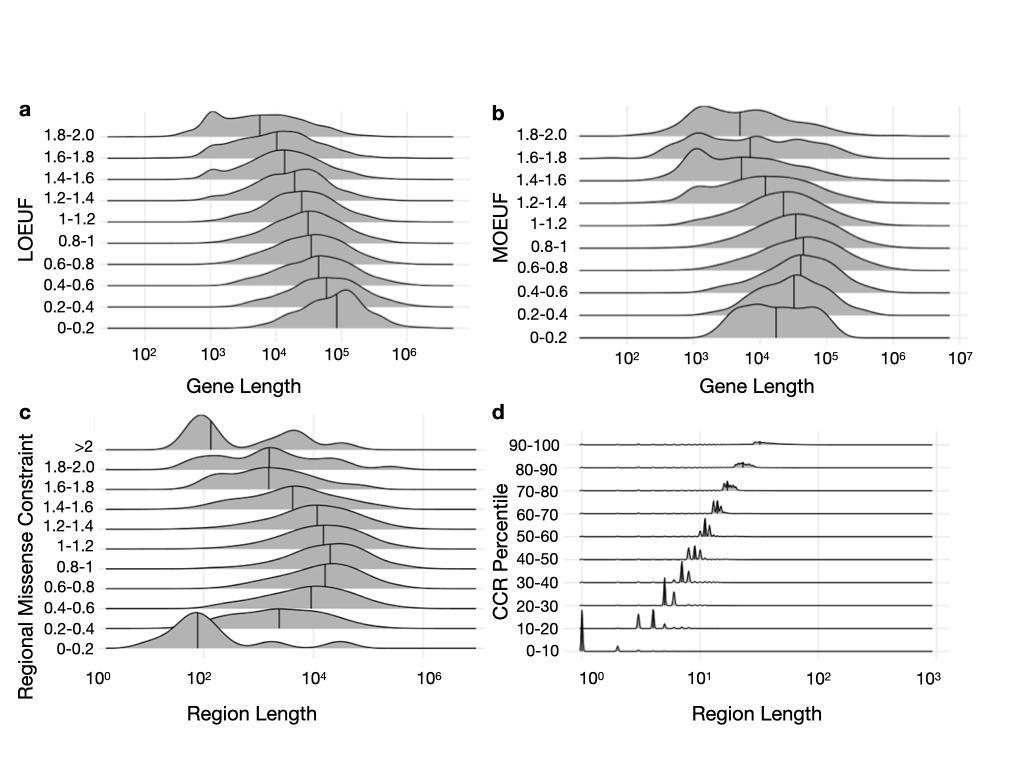
**Fig. S1. The relationship between the length of a coding region and its genic or regional level constraint scores.** We demonstrated the relationship using four existing genic and regional constraint scores: LOEUF[^4^](https://paperpile.com/c/ujbZ45/m5j7I) (a), MOEUF[^4^](https://paperpile.com/c/ujbZ45/m5j7I) (b), Regional Missense Constraint (RMC)[^12^](https://paperpile.com/c/ujbZ45/lc7WD) (c) and CCR[^13^](https://paperpile.com/c/ujbZ45/Xgvmw) (d). The probability density of the length of a coding region and the median (indicated as the vertical line) across different ranges of constraint scores are shown. Constrained bins could be loosely defined as: LOEUF<1, MOEUF<1, RMC<1, CCR Percentile>90. Since these existing metrics measure the constraint signals linearly along the exome, in most cases, their constrained bins include longer coding sequences likely clustered with pathogenic variants than the unconstrained bins. Regions/Genes with short sequences or pathogenic variants sparsely distributed could be missed out as unconstrained.


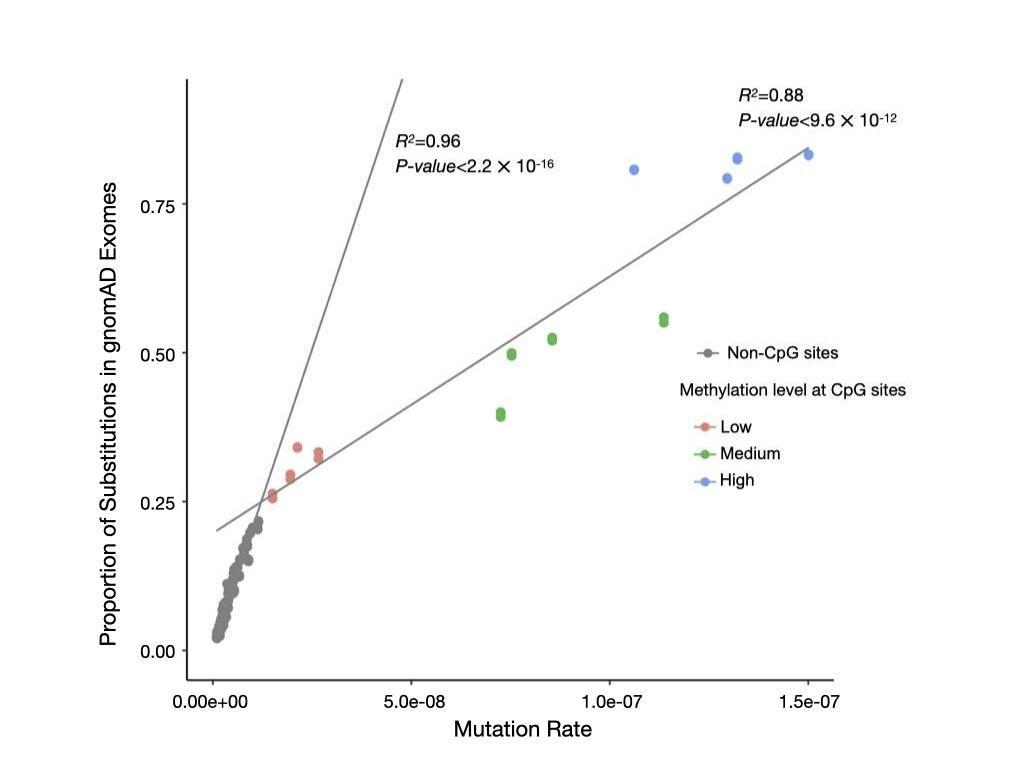


**Fig. S2.** **Calibration of baseline mutation rates to probabilities of neutral substitutions.** Two linear regression models were fitted to predict the proportions of neutral substitutions within the 125,478 exomes from gnomAD: one for CpG sites and the other one for non-CpG sites. This shows that the model is well calibrated for the effect of CpG methylation. In the plot, each dot represents a type of substitution specified by trinucleotide sequence context and methylation level (for CpG sites).


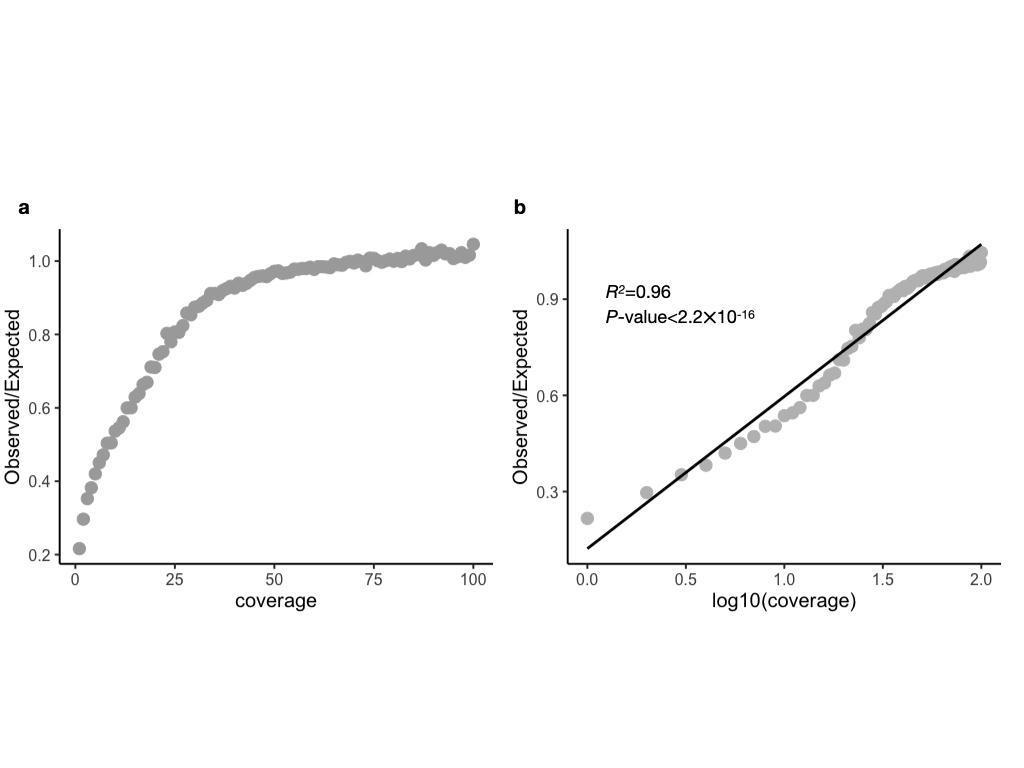


**Fig. S3.** **Calibration of probabilities of neutral substitutions on low-coverage sites (coverage<40).** (a) The relationship between sequencing coverage and Observed/Expected ratios. (b) A linear model is fitted to predict the Observed/Expected ratios given a sequencing coverage on log_10_ scale. The predicted Observed/Expected ratios were used as correction factors to adjust the expected number of variants at low-coverage sites.


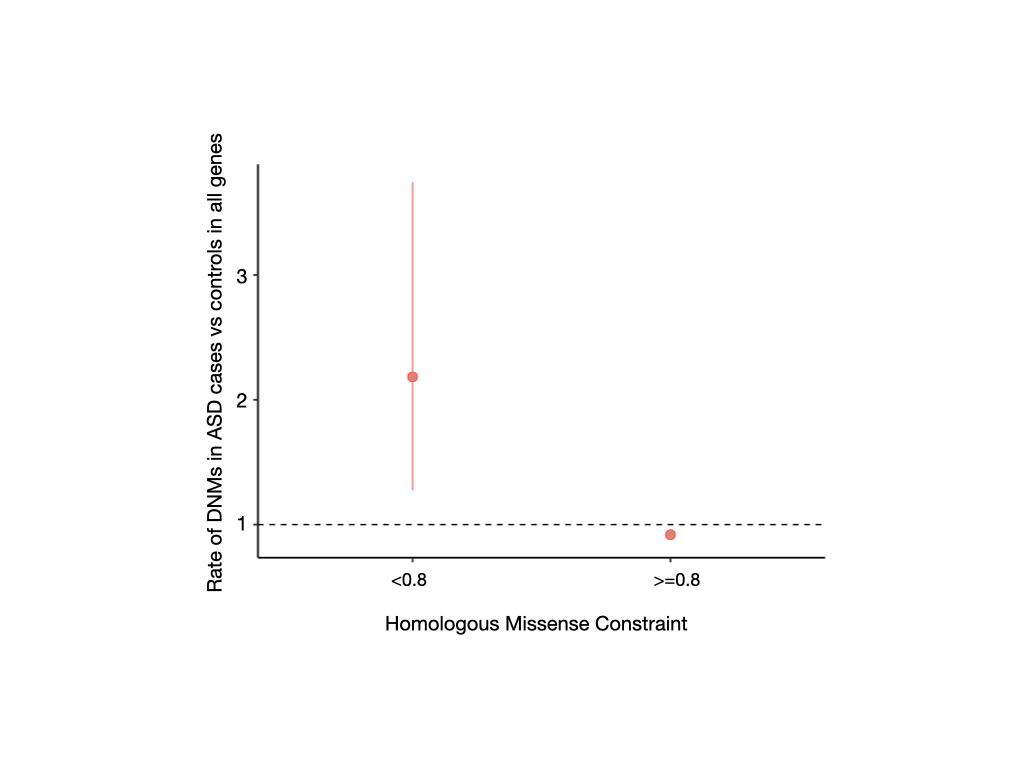


**Fig. S4. Enrichment of constrained missense DNMs in 6,430 patients ascertained with autism spectrum disorders versus 2,179 unaffected controls.**


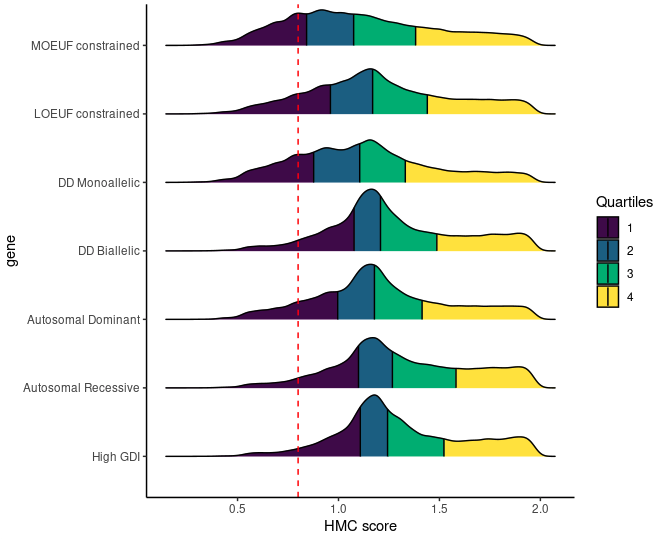


**Fig. S5. Distribution of HMC scores across variants in different gene categories.** The gene categories include MOEUF-constrained genes (MOEUF<0.6), LOEUF-constrained genes (LOEUF<0.6), genes causing developmental disorder via monoallelic and biallelic inheritance mode (with definite confidence from DDG2P panel), autosomal dominant genes, autosomal dominant recessive genes, and genes with high Gene Damage Index[^14^](https://paperpile.com/c/ujbZ45/xtLVf). The red dotted line indicates the HMC-constrained threshold <0.8. Genes with higher selective pressure including MOEUF-constrained genes, LOEUF-constrained genes and DD monoalleic genes tend to have lower HMC scores across variants compared with the other gene categories.


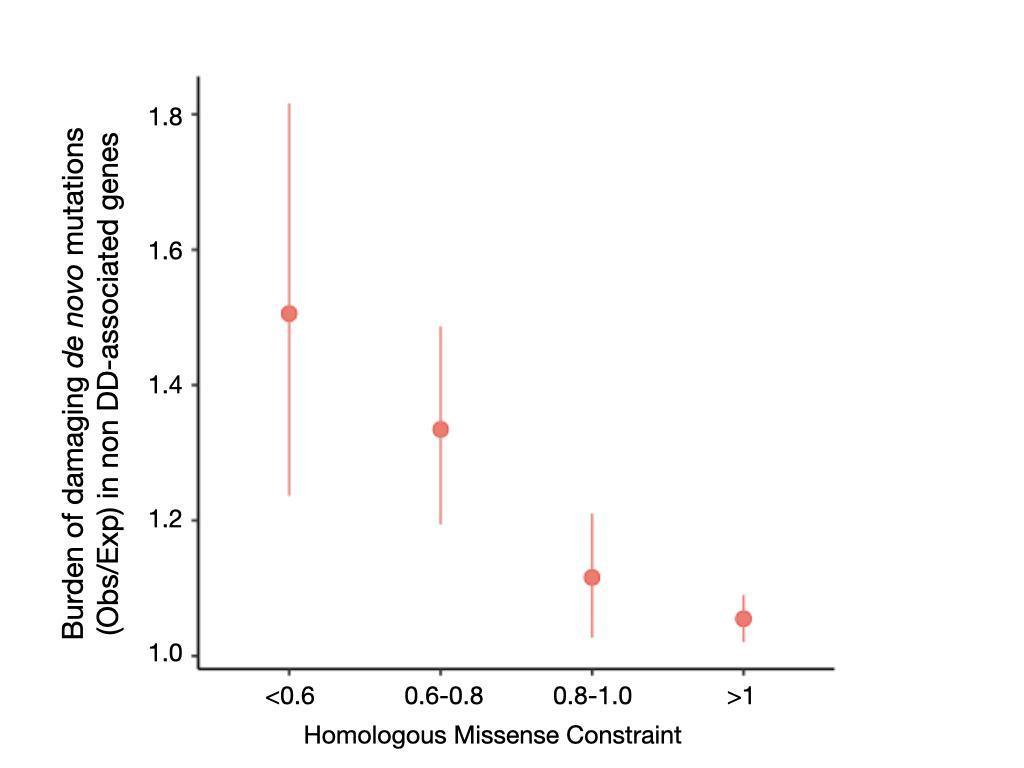


**Fig. S6. Enrichment of constrained missense DNMs of non DD-associated genes in 31K DD trios.** 18,644 genes not considered in diagnostics are defined as non-DD genes as described in the publication of 31K DD cohort[^9^](https://paperpile.com/c/ujbZ45/5RX20).


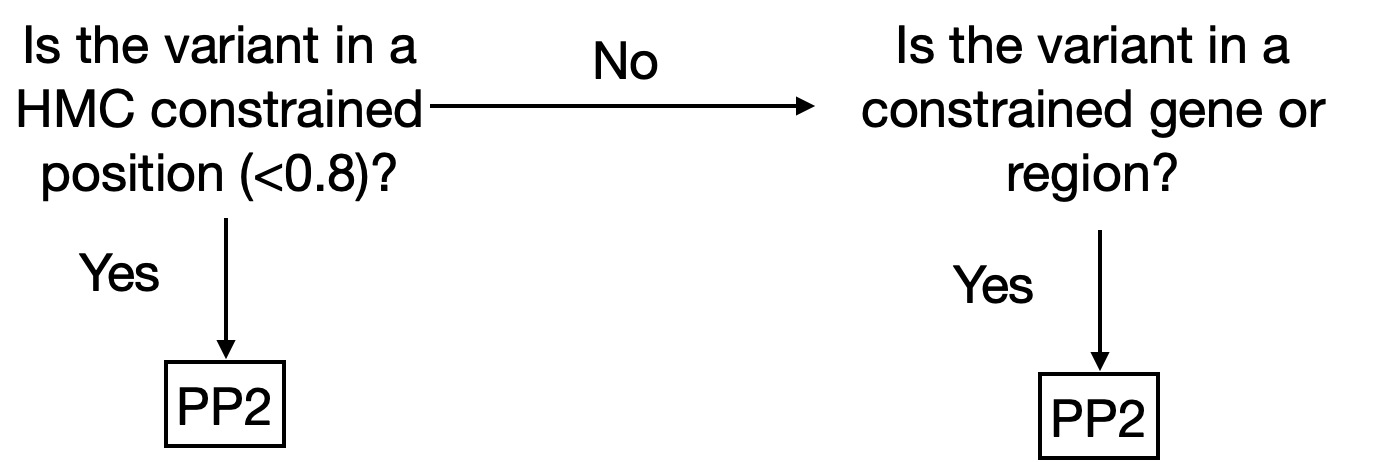


**Fig. S7. Decision tree to use HMC score in a clinical workflow as PP2 (supporting evidence of pathogenicity) following the ACMG guidelines.**


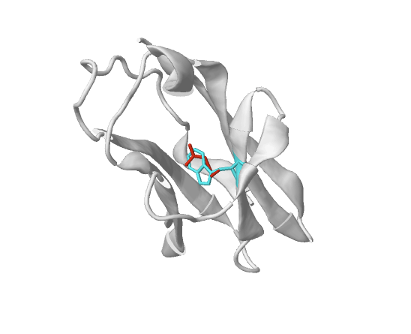


**Fig. S8.** **Structural analysis of TTN p.Trp976Arg missense variant (aqua=wildtype residue; red=mutant residue) using Missense3D**[^15^](https://paperpile.com/c/ujbZ45/WMwOG)**.**

**
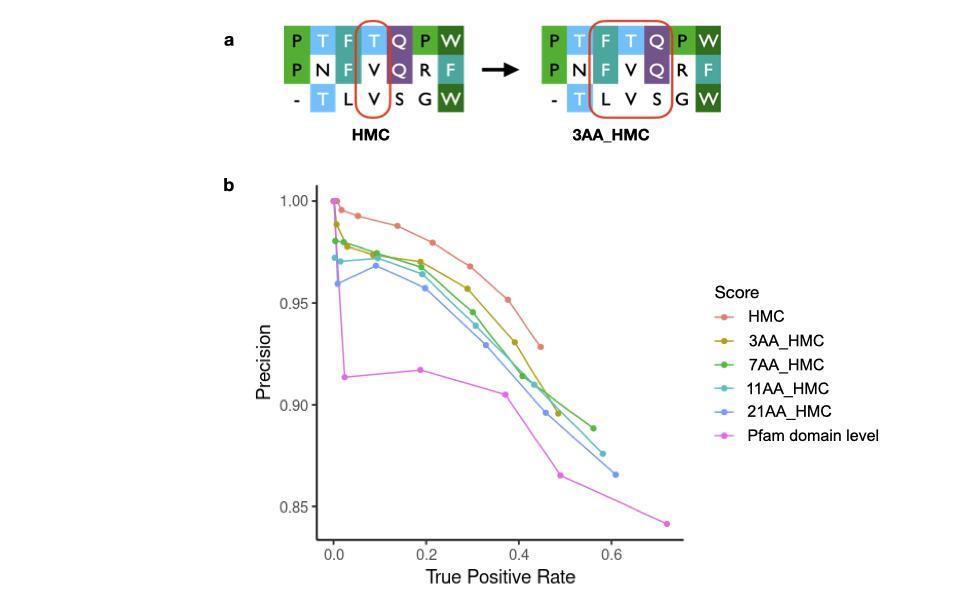
**

**Fig. S9. Exploring alternative genetic constraints measured in Pfam domains.** The following metrics are calculated: HMC (original), 3AA_HMC (Constraint of homologous residues within a sliding window of 3 amino acids, illustrated in **a**), 7AA_HMC, 11AA_HMC, 21AA_HMC (using a sliding window of 7, 11 and 21 amino acids to estimate constraints respectively) and genetic constraint of domain-level. Their performance was compared by classifying ClinVar interpreted data shown as Precision-Recall curves in **b**. HMC has the best precision over all the alternative methods when the constraint scores are under 1.


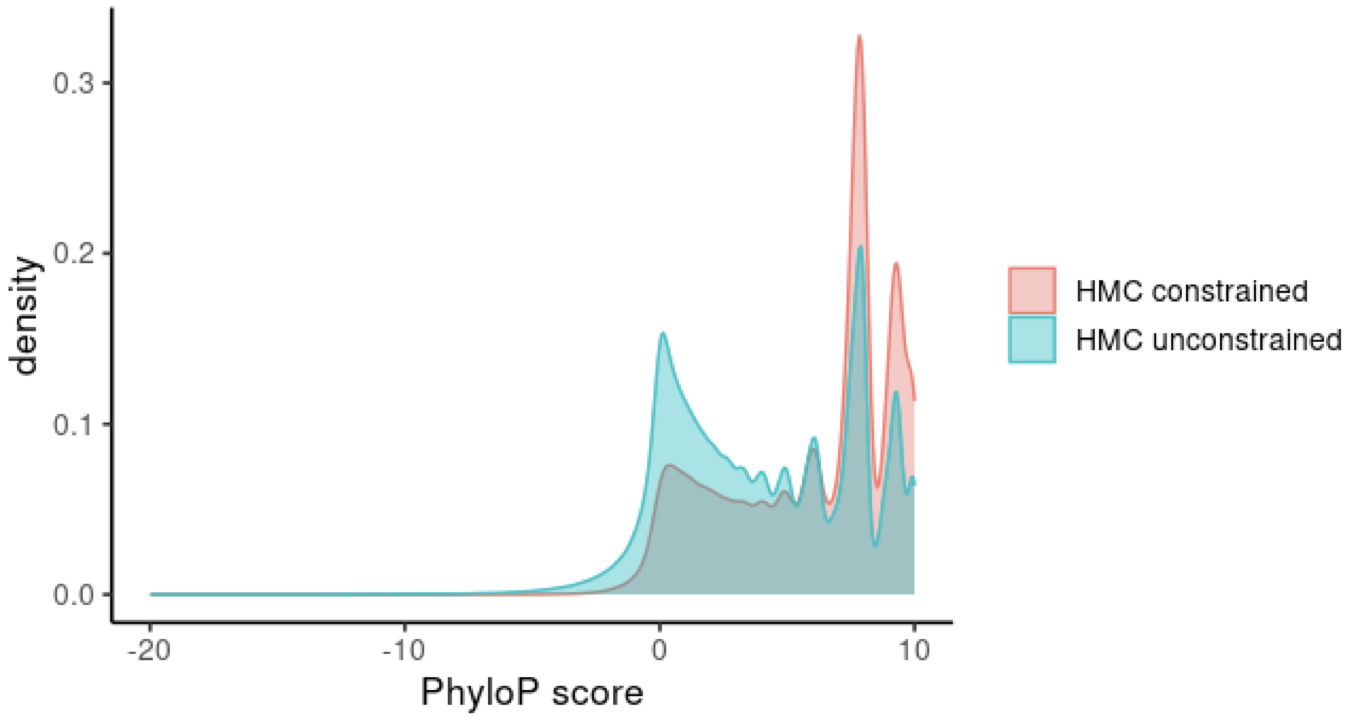


**Fig. S10. Compare the distribution of PhyloP (100way vertebrate) for HMC constrained/unconstrained variants.** The median PhyloP scores for HMC-constrained (HMC<0.8) and unconstrained variants are 7.57 (more conserved) and 4.38 (less conserved) respectively. The Spearman correlation between HMC and PhyloP is -0.16. From the distribution, we can see they don’t always agree with each other thus highlighting the novelty of the HMC score.

## [**Supplementary**](#_30j0zll) **Tables**

**Table S1: Comparing HMC score with MAVE assays across 17 assessable genes.**

| Gene | HMC<0.8  OR  (95% CI) | HMC<1  OR  (95% CI) | Spearman Correlation | AUC | In DDG2P Panel  (Green Rating)* | Allelic Status associated with Developmental Disorder* |
| --- | --- | --- | --- | --- | --- | --- |
| *BRCA1* | 12.67  (7.08-22.68) | 3.89  (2.75-5.50) | 0.20 | 0.62 | TRUE | Biallelic |
| *CBS* | 1.75  (0.58-5.25) | 1.12  (0.57-2.19) | 0.03 | 0.57 | TRUE | Biallelic |
| *PPM1D* | 2.20  (1.16-4.18) | 3.21  (2.32-4.45) | 0.24 | 0.61 | TRUE | Monoallelic |
| *HRAS* | 4.95  (3.67-6.67) | 2.19  (1.41-3.41) | 0.16 | 0.72 | TRUE | Monoallelic |
| *KRAS (Assay type: Cellular Abundance)^+^* | 1.06  (0.76-1.48) | 0.67  (0.43-1.02) | -0.07 | 0.50 | TRUE | Monoallelic |
| *KRAS (Assay type:* RAF1 *binding ^+^* | 9.05  (5.85-14.00) | 6.04  (2.43-15.01) | 0.34 | 0.82 | TRUE | Monoallelic |
| *PSAT1* | 1.20  (0.20-7.23) | 0.26  (0.08-0.81) | 0.05 | 0.52 | TRUE | Biallelic |
| *YAP1* | 34.08  (2.00-580.15) | 1.56  (0.82-2.92) | 0.41 | 0.71 | TRUE | Monoallelic |
| *SUMO1* | 0.82  (0.52-1.30) | 1.44  (0.98-2.11) | 0.07 | 0.51 | FALSE |  |
| *CBX4* | 0.33  (0.01-10.11) | 0.33  (0.01-10.11) | 0.17 | 0.75 | FALSE |  |
| *DNAJA1* | 2.23  (0.04-125.22) | 0.56  (0.01-30.96) | 0.25 | NA | FALSE |  |
| *GRB2* | 1.02  (0.49-2.13) | 2.12  (1.29-3.47) | 0.23 | 0.64 | FALSE |  |
| *KCNH2* | 3.29  (0.66-16.38) | 0.56  (0.01-29.10) | 0.25 | 0.68 | FALSE |  |
| *NUDT15* | 0.32  (0.04-2.69) | 2.42  (1.03-5.70) | 0.10 | 0.54 | FALSE |  |
| *SERPINE1* | 12.31  (0.71-213.55) | 8.33  (2.55-27.22) | 0.15 | 0.59 | FALSE |  |
| *SLC6A4* | 1.71  (1.21-2.42) | 1.64  (1.34-2.01) | 0.11 | 0.56 | FALSE |  |
| *UBE2I* | 1.50  (1.13-2.00) | 1.94  (1.47-2.58) | 0.20 | 0.60 | FALSE |  |

^*^Available from DECIPHER database <https://www.deciphergenomics.org/ddd/ddgenes>

^+^For *KRAS* gene, we chose to use the assay measuring mutational effect on binding to RAF1 given it’s more correlated to HMC assuming it reflects more on reproductive fitness effect.

**Table S2: Comparison of DNM burdens of constrained and unconstrained missense variants in the upgraded and original DeNovoWEST tests in the 31K DD cohort.** In DeNovoWEST, missense variants are subsetted based on missense constrained information, CADD scores and S_het_ values (“estimated selective effect of heterozygous PTVs on gene level”) and scored according to observed DNM burdens (obs/exp ratio) in the 31K DD cohort. Compared with original version of DeNovoWEST, after we incorporated HMC to score missense variants, constrained missense variants have increased enrichments while unconstrained missense variants have decreased enrichments, indicating that HMC improved the discrimination of missense variants associated with diseases.

|  | DNM burden and 95%CI after upgrading | DNM burden and 95%CI  in the original publication[^9^](https://paperpile.com/c/ujbZ45/5RX20) |
| --- | --- | --- |
| Constrained missense variants | 2.661 [2.581-2.743] | 2.597 [2.513-2.684] |
| Unconstrained missense variants | 1.127 [1.113-1.141] | 1.145 [1.130-1.159] |

**Table S3: Newly-significant DD-associated genes found in the full cohort of 31K DD trios.**

| Gene | *P*-value in original DeNovoWEST | *P*-value in updated DeNovoWEST | Number of PTVs | Number of missense variants  (constrained/total) | DDG2P Confidence in Monoallelic mode^*^ (version 2021.12.12) |
| --- | --- | --- | --- | --- | --- |
| *BMPR2* | 6.25e-06 | 9.59e-10 | 2 | 3/4 | No |
| *DHX30* | 2.62e-06 | 1.63e-08 | 0 | 7/8 | Probable |
| *GABBR2* | 3.56e-06 | 1.78e-08 | 1 | 4/8 | Probable |
| *KCNC2* | 3.12e-03 | 2.64e-07 | 0 | 3/4 | No |
| *MSI1* | 2.74e-05 | 6.53e-07 | 2 | 3/5 | Possible |
| *RAB5C* | 2.12e-04 | 1.13e-08 | 1 | 2/5 | No |
| *SATB1* | 2.22e-06 | 1.65e-11 | 3 | 4/7 | Confirmed |

^*^ - The terminology used to describe gene-disease validity is explained here https://www.ebi.ac.uk/gene2phenotype/terminology

**Table S4: Newly-significant DD-associated genes found in the undiagnosed cohort of 24K DD trios.**

| Gene | *P*-value in original DeNovoWEST | *P*-value in updated DeNovoWEST | Number of PTVs | Number of missense variants  (constrained/total) | Status in DDG2P in Monoallelic mode (version 2021.12.12) |
| --- | --- | --- | --- | --- | --- |
| *BMPR2* | 2.44e-06 | 1.26e-10 | 1 | 3/4 | No |
| *GABBR2* | 3.56e-06 | 8.34e-09 | 0 | 4/7 | Probable |
| *MSI1* | 6.47e-06 | 1.16e-07 | 2 | 3/5 | Possible |
| *RAB5C* | 1.19e-03 | 7.07e-08 | 1 | 2/4 | No |

**Resource Availability**

External data used in the study were obtained from the following approaches/URLs:

1. The IDs of RefSeq Select Transcripts were downloaded from the UCSC Genome Browser using the Table Browser tool (downloaded date: Nov 28th 2020; options used: group - “Genes and Gene Predictions”, track - “NCBI RefSeq”, table - “RefSeq Select”);
2. ClinVar, https://ftp.ncbi.nlm.nih.gov/pub/clinvar/vcf_GRCh37/archive_2.0/2020/clinvar_20201114.vcf.gz (downloaded at Nov 2020) ;
3. Developmental Disorder Genotype-Phenotype Database (DDG2P), https://www.deciphergenomics.org/ddd/ddgenes (version 2021.11.05);
4. The 31K DD trio data and the original DeNovoWEST, <https://github.com/HurlesGroupSanger/DeNovoWEST>;
5. gnomAD exome, v2.1.1, <https://gnomad.broadinstitute.org/downloads>;
6. The hypertrophic cardiomyopathy case series curated by the SHaRe Consortium (data release 2019Q3): https://github.com/ImperialCardioGenetics/CardioBoost_manuscript/tree/master/data/cardiomyopathy/share_variant_count.RData;
7. CCR score, <https://github.com/quinlan-lab/ccr>;
8. RMC and MPC score, https://storage.googleapis.com/gcp-public-data--gnomad/legacy/exac_browser/regional_missense_constraint.tsv, <ftp://ftp.broadinstitute.org/pub/ExAC_release/release1/regional_missense_constraint/fordist_constraint_official_mpc_values.txt.gz>;
9. M-CAP score, http://bejerano.stanford.edu/mcap/ (v1.4); REVEL score, <https://sites.google.com/site/revelgenomics>;
10. CADD, https://cadd.gs.washington.edu/download; para_zscore, https://zenodo.org/record/3582386#.YYxNXb1_rSw (version 9).
11. Known autosomal dominant, autosomal recessive disease genes: downloaded from https://github.com/macarthur-lab/gene_lists

**References in Supplementary Information**

1. [El-Gebali, S. *et al.* The Pfam protein families database in 2019. *Nucleic Acids Res.* **47**, D427–D432 (2019).](http://paperpile.com/b/ujbZ45/acs6Q)

2. [McLaren, W. *et al.* The Ensembl Variant Effect Predictor. *Genome Biol.* **17**, 122 (2016).](http://paperpile.com/b/ujbZ45/dsXyo)

3. [Lek, M. *et al.* Analysis of protein-coding genetic variation in 60,706 humans. *Nature* **536**, 285–291 (2016).](http://paperpile.com/b/ujbZ45/j9ZTu)

4. [Karczewski, K. J. *et al.* The mutational constraint spectrum quantified from variation in 141,456 humans. *Nature* **581**, 434–443 (2020).](http://paperpile.com/b/ujbZ45/m5j7I)

5. [Landrum, M. J. *et al.* ClinVar: improving access to variant interpretations and supporting evidence. *Nucleic Acids Res.* **46**, D1062–D1067 (2018).](http://paperpile.com/b/ujbZ45/1Ne5a)

6. [Satterstrom, F. K. *et al.* Large-Scale Exome Sequencing Study Implicates Both Developmental and Functional Changes in the Neurobiology of Autism. *Cell* **180**, 568–584.e23 (2020).](http://paperpile.com/b/ujbZ45/hrQrc)

7. [Samocha, K. E. *et al.* A framework for the interpretation of de novo mutation in human disease. *Nat. Genet.* **46**, 944–950 (2014).](http://paperpile.com/b/ujbZ45/Myl9r)

8. [Ware, J. S., Samocha, K. E., Homsy, J. & Daly, M. J. Interpreting de novo Variation in Human Disease Using denovolyzeR. *Curr. Protoc. Hum. Genet.* **87**, 7.25.1–7.25.15 (2015).](http://paperpile.com/b/ujbZ45/7CTXj)

9. [Kaplanis, J. *et al.* Evidence for 28 genetic disorders discovered by combining healthcare and research data. *Nature* **586**, 757–762 (2020).](http://paperpile.com/b/ujbZ45/5RX20)

10. [Firth, H. V., Wright, C. F. & DDD Study. The Deciphering Developmental Disorders (DDD) study. *Dev. Med. Child Neurol.* **53**, 702–703 (2011).](http://paperpile.com/b/ujbZ45/FX0wS)

11. [Notin, P. *et al.* ProteinGym: Large-scale benchmarks for protein fitness prediction and design. *Adv. Neural Inf. Process. Syst.* (2023).](http://paperpile.com/b/ujbZ45/UGpd0)

12. [Samocha, K. E. *et al.* Regional missense constraint improves variant deleteriousness prediction. Preprint at https://doi.org/](http://paperpile.com/b/ujbZ45/lc7WD)[10.1101/148353](http://dx.doi.org/10.1101/148353)[.](http://paperpile.com/b/ujbZ45/lc7WD)

13. [Havrilla, J. M., Pedersen, B. S., Layer, R. M. & Quinlan, A. R. A map of constrained coding regions in the human genome. *Nat. Genet.* **51**, 88–95 (2019).](http://paperpile.com/b/ujbZ45/Xgvmw)

14. [Itan, Y. *et al.* The human gene damage index as a gene-level approach to prioritizing exome variants. *Proc. Natl. Acad. Sci. U. S. A.* **112**, 13615–13620 (2015).](http://paperpile.com/b/ujbZ45/xtLVf)

15. [Ittisoponpisan, S. *et al.* Can Predicted Protein 3D Structures Provide Reliable Insights into whether Missense Variants Are Disease Associated? *J. Mol. Biol.* **431**, 2197–2212 (2019).](http://paperpile.com/b/ujbZ45/WMwOG)
